# Supplementary material for: A versatile toolkit for CRISPR-Cas13-based RNA manipulation in Drosophila
Source: Genome Biol. 2020 Nov 17;21:279. doi: 10.1186/s13059-020-02193-y (PMC7670108; doi:10.1186/s13059-020-02193-y)
Supplement: Supplementary file 1 — Additional file 1. Supplementary figures S1-S8. [file 13059_2020_2193_MOESM1_ESM.pdf]

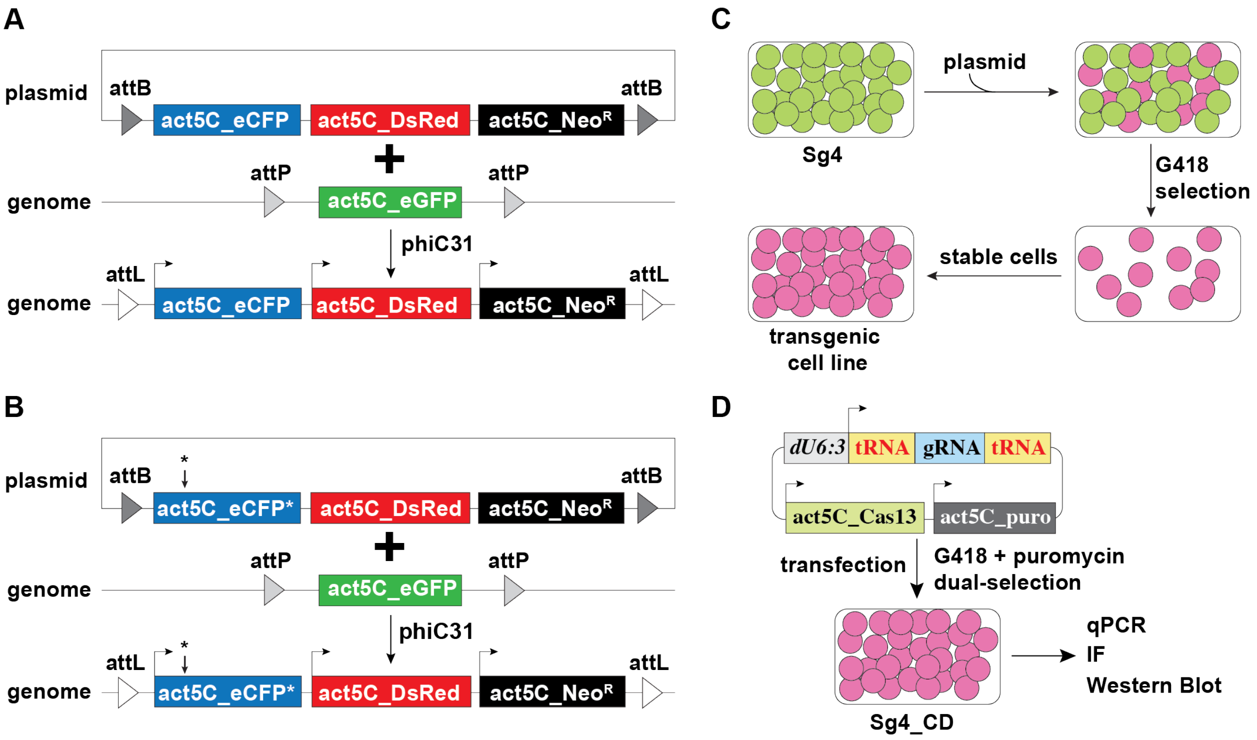

Fig S1: Schematic of transgenic cell culture and *in vitro* study. (A) Generation of Sg4_CD cell line that expresses *eCFP*, *DsRed*, and Neo^R genes under independent *actin5C* (*ac5*) promoters. **(B)** Generation of Sg4* cell line that expresses mutant *eCFP**, *DsRed*, and Neo^R genes under independent *actin5C* (*ac5*) promoters. **(C)** Establishment of the transgenic cell line. Two days after transfection, cells were supplemented with geneticin (G418). Cells without transfected plasmid will be eliminated eventually, leaving cells with successful integration. Cells were passaged for at least four rounds, and integration was confirmed via sequencing. **(D)** Schematic of pC13cr01 vector activity. Upon transfection with the pC13cr01 vector, the cells were selected with geneticin and puromycin to eliminate untransfected cells. Seven days after transfection, cells were collected for later study.

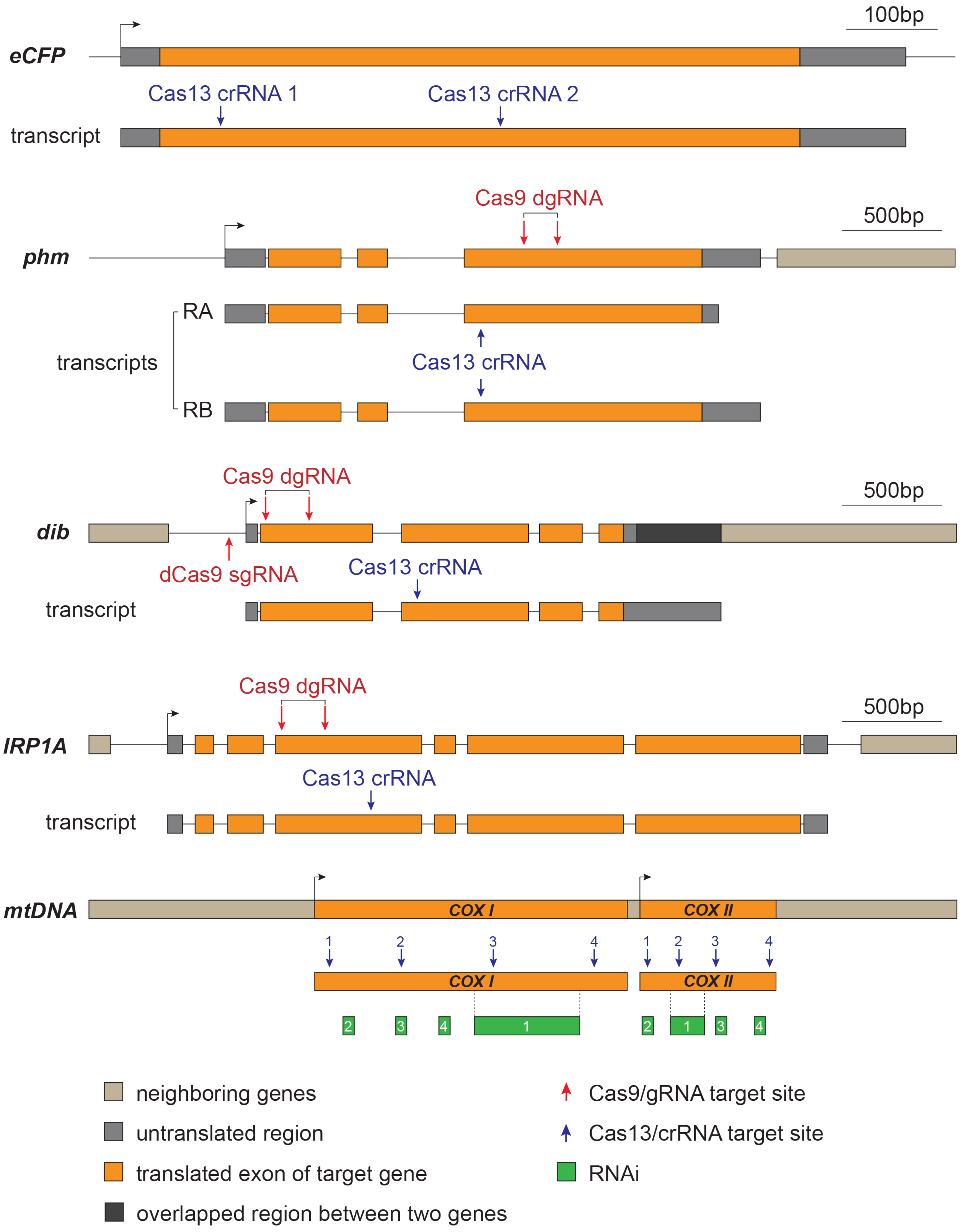
100bp
eCFP
Cas13 crRNA 1
Cas13 crRNA 2
transcript
Cas9 dgRNA
500bp
phm
RA
transcripts
Cas13 crRNA
RB
Cas9 dgRNA
500bp
dib
dCas9 sgRNA
Cas13 crRNA
transcript
Cas9 dgRNA
500bp
IRP1A
Cas13 crRNA
transcript
mtDNA
COX I
COX II
1
2
3
4
1
2
3
4
COX I
COX II
2
3
4
1
2
1
3
4
neighboring genes
untranslated region
translated exon of target gene
overlapped region between two genes
Cas9/gRNA target site
Cas13/crRNA target site
RNAi

Fig S2: Target sites of crRNAs. For *in vitro* evaluation, we tested *eCFP* expression and two mitochondrial-encoded transcripts, *COXI* and *COXII*. For the *in vivo* approach, we tested two genes that encode enzymes acting as ecdysteroid-synthesizing enzymes in the *Drosophila* prothoracic gland, *phantom* (*phm*) and *disembodied* (*dib*) and a gene involved in cellular iron homeostasis, namely *iron regulatory protein 1A* (*IRP1A)*. Shown here are the target sites for crRNA (Cas13-compatible, blue), gRNA (Cas9-compatible, red), and RNAi (green) for transcripts we tested.

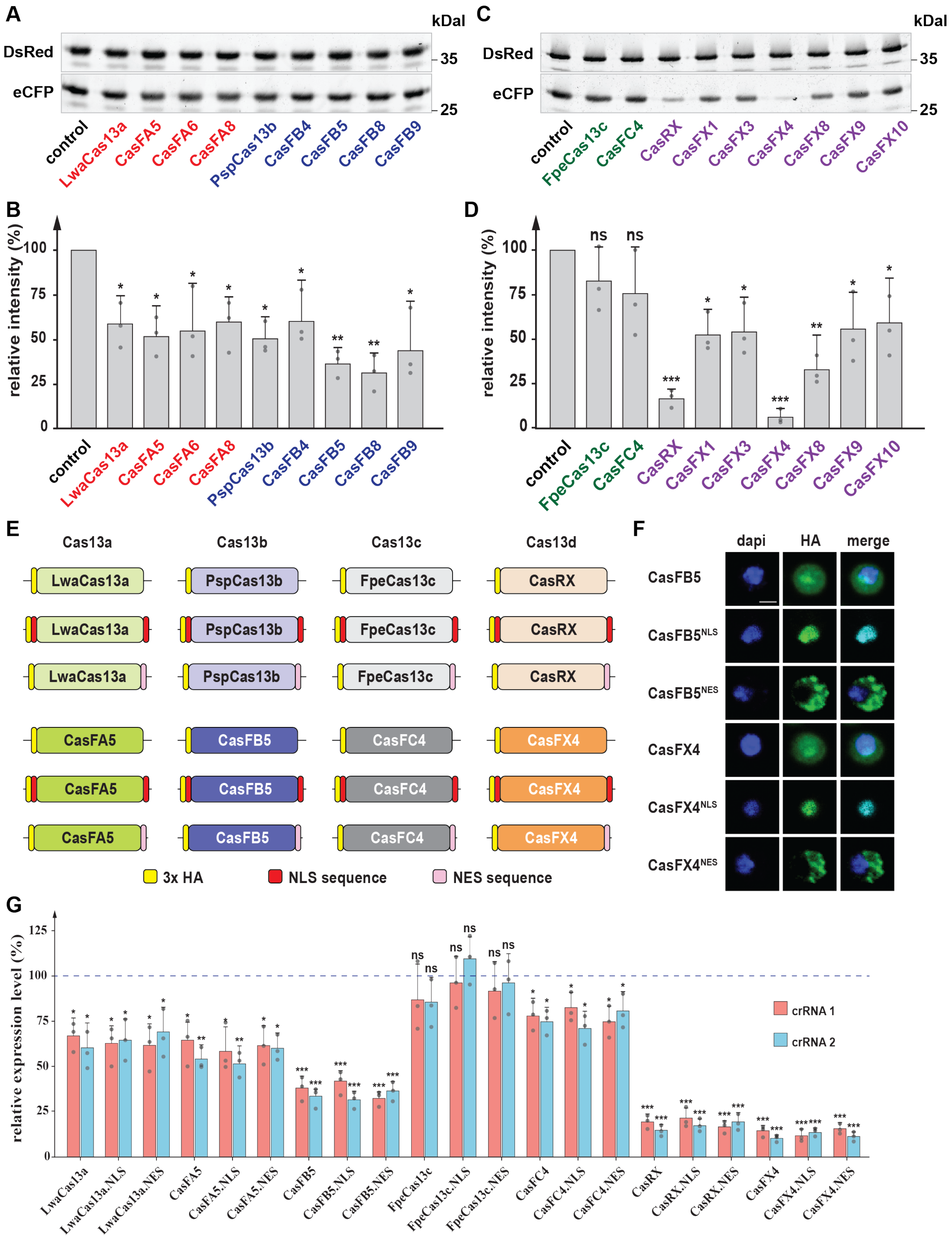
A
kDal
DsRed
35
eCFP
25
control
LwaCas13a
CasFA5
CasFA6
CasFA8
PspCas13b
CasFB4
CasFB5
CasFB8
CasFB9
C
kDal
DsRed
35
eCFP
25
control
FpeCas13c
CasFC4
CasRX
CasFX1
CasFX3
CasFX4
CasFX8
CasFX9
CasFX10
B
relative intensity (%)
D
relative intensity (%)
ns
E
Cas13a
Cas13b
Cas13c
Cas13d
LwaCas13a
PspCas13b
FpeCas13c
CasRX
CasFA5
CasFB5
CasFC4
CasFX4
3x HA
NLS sequence
NES sequence
F
dapi
HA
merge
CasFB5
CasFB5NLS
CasFB5NES
CasFX4
CasFX4NLS
CasFX4NES
G
relative expression level (%)
crRNA 1
crRNA 2
LwaCas13a
LwaCas13a.NLS
LwaCas13a.NES
CasFA5
CasFA5.NLS
CasFA5.NES
CasFB5
CasFB5.NLS
CasFB5.NES
FpeCas13c
FpeCas13c.NLS
FpeCas13c.NES
CasFC4
CasFC4.NLS
CasFC4.NES
CasRX
CasRX.NLS
CasRX.NES
CasFX4
CasFX4.NLS
CasFX4.NES

Fig S3: Evaluation of *Drosophila* codon-optimized Cas13 variants. (A-D) Western blotting of eCFP in samples that were treated with Cas13 variants that showed the highest efficiency in qPCR experiments. Band intensities were quantified with ImageJ and normalized to samples treated with blank crRNA. * = p-value < 0.05, ** = p-value < 0.01, *** = p - value < 0.001, p-values based on Dunnett's *post-hoc* test, error bars represent standard error. **(E)** Schematic of Cas13 variants with different signaling sequences, including the nuclear localization signal (NLS) and the nuclear export signal (NES). **(F)** Subcellular localization of CasFB5 and CasFX4 variants in the presence or absence of NLS and NES. Nuclei were stained with dapi (blue) and Cas13 polypeptides were stained with anti-HA antibody (green). Scale bar = 50 µm. **(G)** Evaluation of NLS and NES on Cas13 efficiency via qPCR. Each Cas13 variant was either fused with an NLS or NES and tested for their interference efficiency on eCFP expression. Data were normalized to samples treated with blank crRNA (blue dotted line = 1). * = p-value < 0.05, ** = p-value < 0.01, *** = p-value < 0.001, ns = not significant, p-values based on Student t-tests, error bars represent 95% confidence intervals.

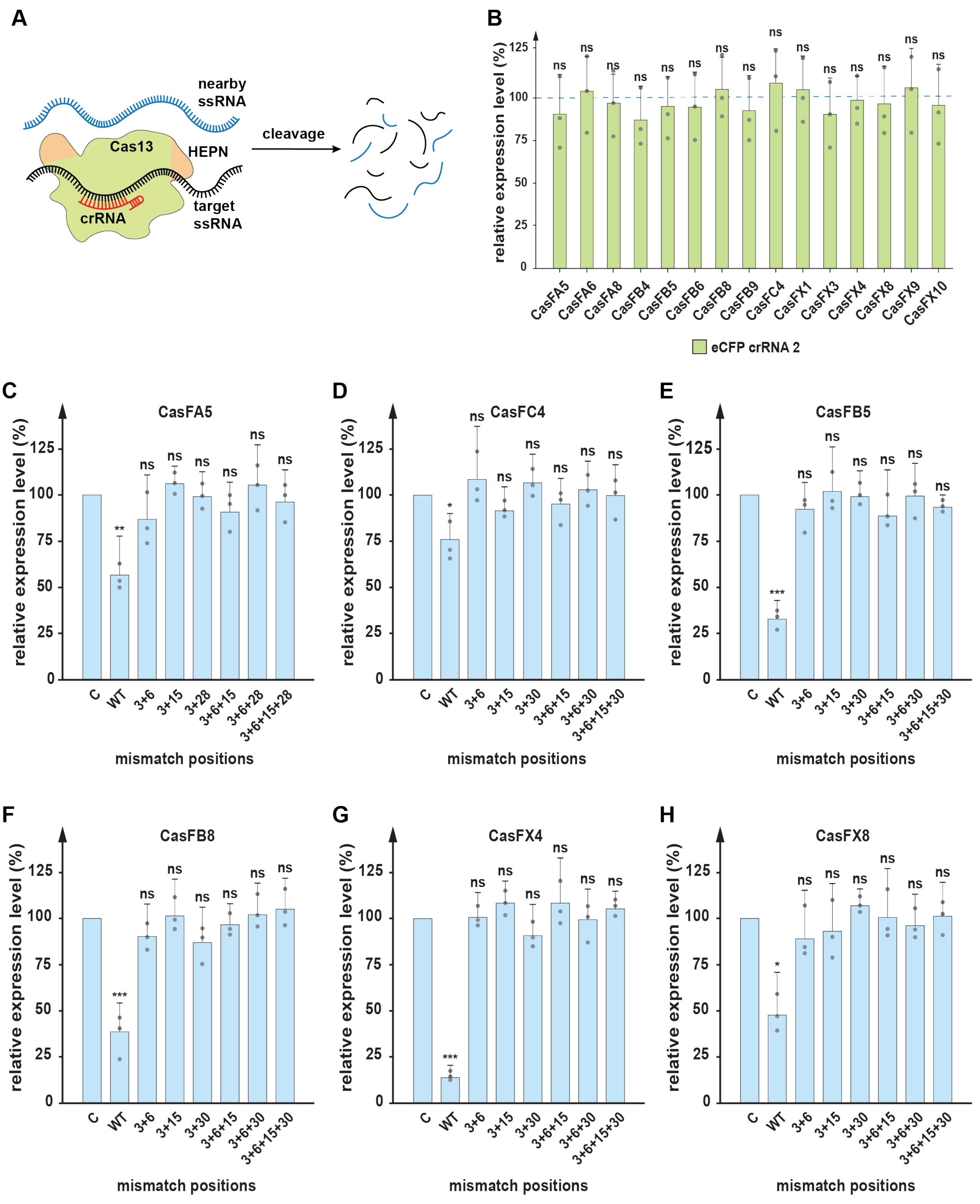

A
nearby ssRNA
Cas13
HEPN
crRNA
target ssRNA
cleavage
B
relative expression level (%)
CasFA5 CasFA6 CasFA8 CasFB4 CasFB5 CasFB6 CasFB8 CasFB9 CasFC4 CasFX1 CasFX3 CasFX4 CasFX8 CasFX9 CasFX10
eCFP crRNA 2
C
CasFA5
D
CasFC4
E
CasFB5
F
CasFB8
G
CasFX4
H
CasFX8
mismatch positions

Fig S4: Collateral activity and specificity evaluation of Cas13 variants. (A) Schematic of collateral activity in Cas13. Overall, once a complex is formed with its crRNA, and upon binding to target transcripts, Cas13 will undergo a conformational change, which results in the exposure of two nuclease domains (HEPN). This exposure allows the domains to interact with nearby non-specific transcripts and results in their degradation. **(B)** Relative expression of *DsRed* in samples treated with Cas13/crRNA against eCFP. The *act5*C promoter drives DsRed. It is believed that the DsRed transcript is present in high amounts, and is more likely to interact with Cas13. Therefore, if the collateral activity is an issue, we would be expected that DsRed transcript levels are affected. Data were normalized to samples treated with blank crRNA (blue dotted line = 1). * = p-value < 0.05, ** = p-value < 0.01, *** = p-value < 0.001, ns = not significant, error bars represent 95% confidence intervals. **(C-H)** Relative expression levels of eCFP that were exposed to different Cas13 variants and crRNAs carrying different combinations of mismatches along the eCFP crRNA 2. Data were normalized to samples treated with blank crRNA (control = C). eCFP expression level in Cas13/wild-type (WT) crRNA samples were also included as a reference for changes. * = p-value < 0.05, ** = p-value < 0.01, *** = p-value < 0.001, ns = not significant, p-values based on Dunnett's *post-hoc* test, error bars represent 95% confidence intervals.

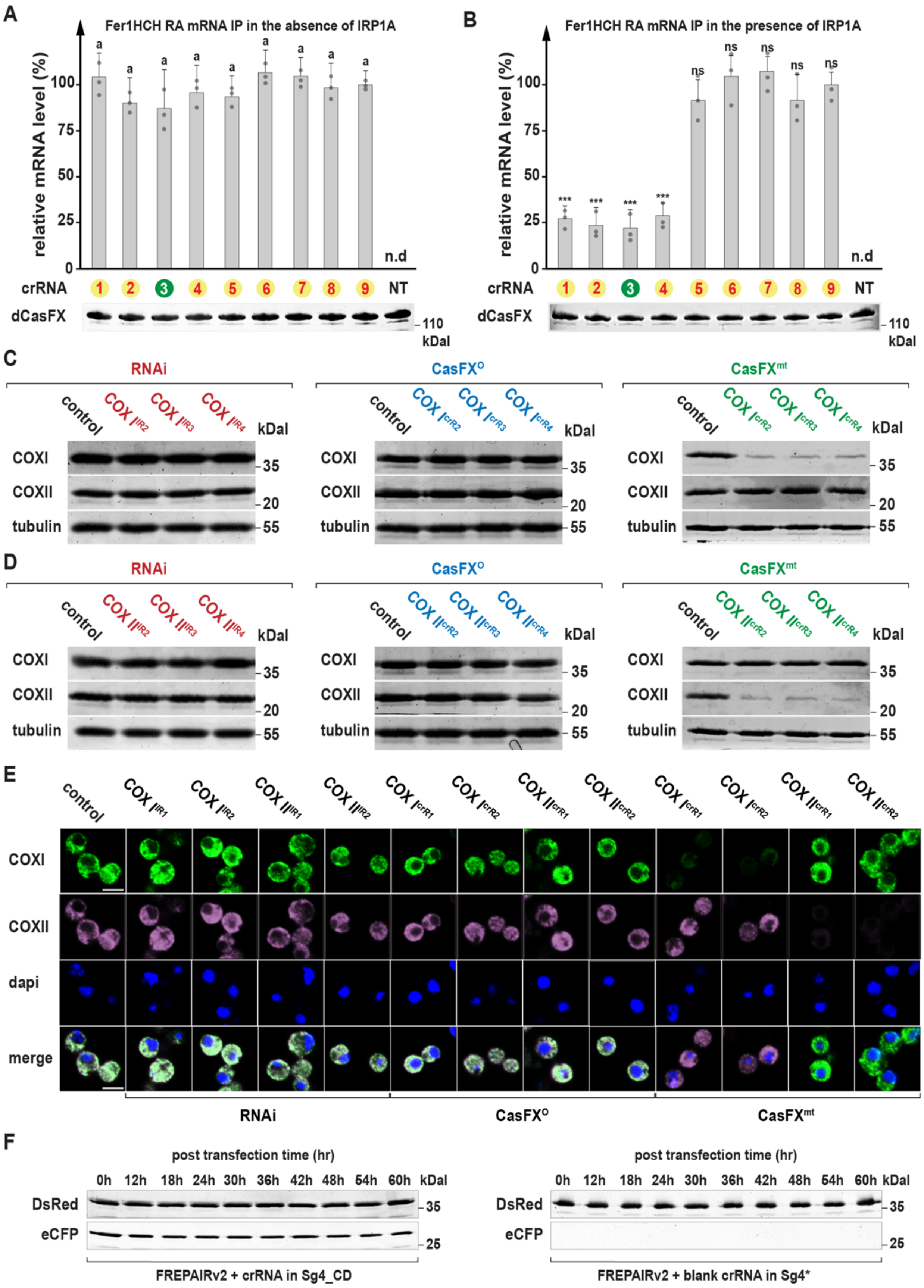
A
Fer1HCH RA mRNA IP in the absence of IRP1A
relative mRNA level (%)
crRNA
1 2 3 4 5 6 7 8 9 NT
n.d
dCasFX
110 kDal
B
Fer1HCH RA mRNA IP in the presence of IRP1A
relative mRNA level (%)
crRNA
1 2 3 4 5 6 7 8 9 NT
n.d
dCasFX
110 kDal
C
RNAi
CasFX°
CasFXmt
control
COX I
COX II
tubulin
kDal
D
RNAi
CasFX°
CasFXmt
control
COXI
COXII
tubulin
kDal
E
control
COXI
COXII
dapi
merge
RNAi
CasFX°
CasFXmt
F
post transfection time (hr)
0h 12h 18h 24h 30h 36h 42h 48h 54h 60h kDal
DsRed
eCFP
FREPAIRv2 + crRNA in Sg4_CD
FREPAIRv2 + blank crRNA in Sg4*

Fig S5: Evaluation of modified CasFX for different approaches. (A-B) Relative *Fer1HCH-RA* mRNA amount that was pulled down by the dCasFX/crRNA complex. **(A)** dCasFX and the crRNA targeting *Fer1HCH-RA* mRNA (Figure 4E) were transfected together into one sample, while *Fer1HCH-RA* was transfected into a different sample of cells. The two samples were lysed and combined, followed by immunoprecipitation (IP) of dCasFX via its added HA tag to test for the presence of *Fer1HCH-RA* mRNA. Results were analyzed by one-way analysis of variance (ANOVA) followed Tukey HSD (HSD = honestly significant difference) *post-hoc* test: groups with different letters are statistically different ($p \leq 0.05$) and groups with the same letters are statistically equal ($p \leq 0.05$), error bars represent 95% confidence intervals. (**B)** dCasFX and crRNA targeting *Fer1HCH-RA* mRNA (Figure 4E) were transfected together into one sample. *Fer1HCH-RA* and IRP1A^{C450S}, the constitutively RNA-binding form of IRP1A that interacts with the iron-responsive element (IRE) in the *Fer1HCH-RA* mRNA, were transfected together into a separate batch of cells. The two samples were lysed and combined, followed by immunoprecipitation (IP) of dCasFX via its attached HA tag to test for the presence of IRP1A in the pull-down assay (Figure 4E) and *Fer1HCH-RA* transcript. Results were analyzed by one-way analysis of variance (ANOVA) followed Tukey HSD (HSD = honestly significant difference) *post-hoc* test: groups with different letters are statistically different ($p \leq 0.05$) and groups with the same letters are statistically equal ($p \leq 0.05$), error bars represent 95% confidence intervals. **(C-D)** Western blotting of COXI and COXII targeted by either RNAi, CasFX^{O}, or CasFX^{mt}. **(E)** Immunofluorescence of COXI and COXII targeted by two independent RNAi constructs, CasFX^{O} or CasFX^{mt}. Nuclei were stained with DAPI (blue), COXI was stained with anti-COXI antibody (cyan), and COXII was stained with anti-COXII antibody (red). Scale bar = 50 µm. (**F)**

Western blotting of wild-type eCFP or mutant eCFP* with blank crRNA under the same condition as FREPAIRv2.

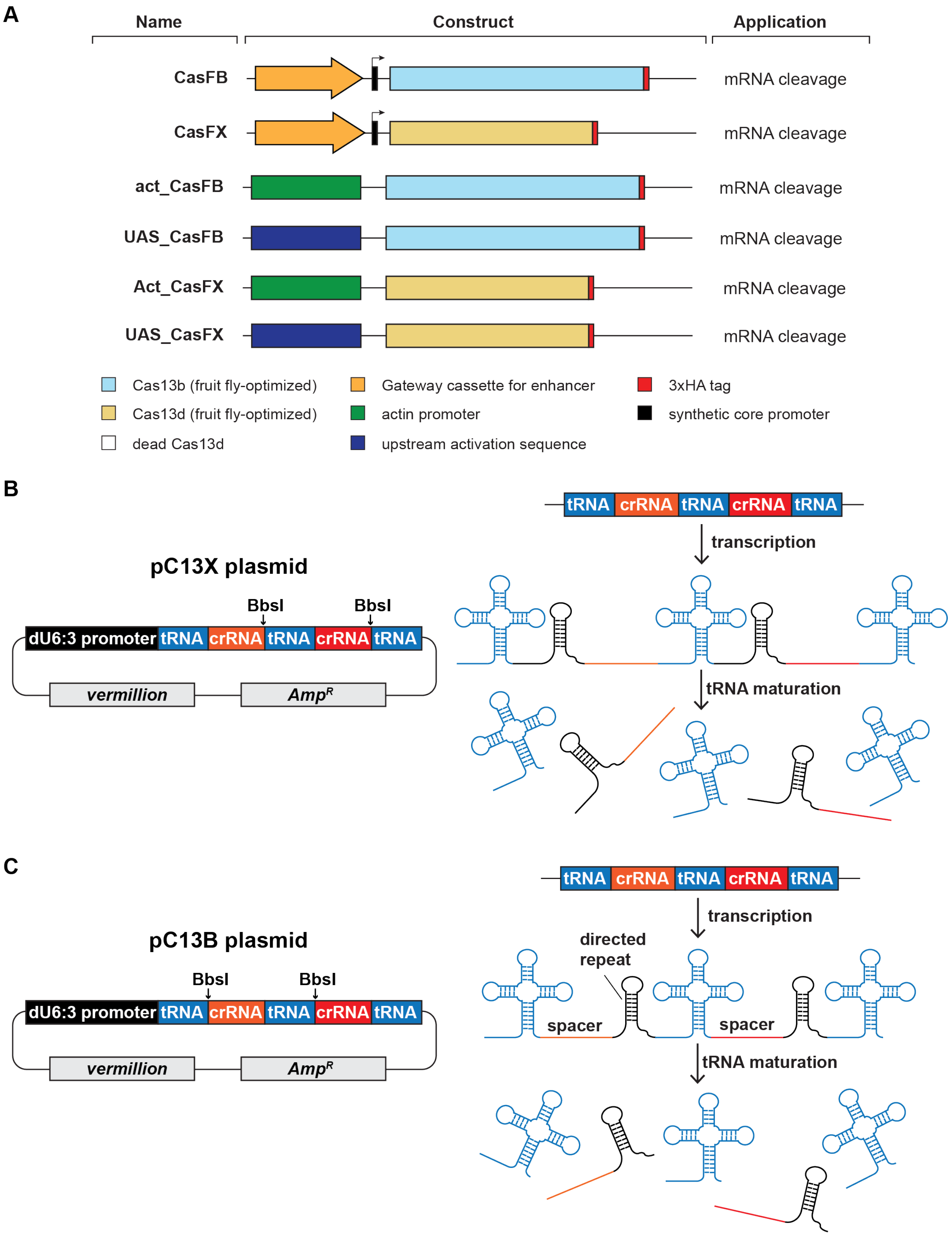
A
Name
Construct
Application
CasFB
mRNA cleavage
CasFX
mRNA cleavage
act_CasFB
mRNA cleavage
UAS_CasFB
mRNA cleavage
Act_CasFX
mRNA cleavage
UAS_CasFX
mRNA cleavage
Cas13b (fruit fly-optimized)
Cas13d (fruit fly-optimized)
dead Cas13d
Gateway cassette for enhancer
actin promoter
upstream activation sequence
3xHA tag
synthetic core promoter
B
pC13X plasmid
BbsI
BbsI
dU6:3 promoter tRNA crRNA tRNA crRNA tRNA
vermillion
AmpR
tRNA crRNA tRNA crRNA tRNA
transcription
tRNA maturation
C
pC13B plasmid
BbsI
BbsI
dU6:3 promoter tRNA crRNA tRNA crRNA tRNA
vermillion
AmpR
tRNA crRNA tRNA crRNA tRNA
transcription
directed repeat
spacer
spacer
tRNA maturation

Fig S6: CRISPR/Cas13 transgenes and crRNA vectors for *in vivo* RNA targeting. (A) Collection of Cas13 transgenes. The general Cas13 collection is composed of a *mini-white* gene as a marker, a PhiC31 integrase-compatible *attB* site, and the *bla* coding sequence to mediate ampicillin resistance and a synthetic core promoter. Shown here are the gateway cassette for an enhancer of choice, and the Cas13 variants. The gateway cassette allows using LR Clonase-based recombination (ThermoFisher) to insert enhancer/promoter regions to drive tissue-specific *Cas9* expression. The *act-Cas13* transgenes drive the expression of *Cas13* via *actin 5C* (*ac5*) promoter while the *UAS-Cas13* transgenes allow tissue-specific expression of *Cas13* via the Gal4/UAS system. In all cases, Cas13 variants were fused with a 3xHA epitope tag at the C-terminal end. **(B)** Collection of Cas13-compatible crRNA vectors. pC13X is compatible with CasF, whereas pC13B is designed for CasFB. Both vectors carry a *vermillion* marker, a PhiC31 integrase-compatible *attB* site, and the *bla* coding sequence to mediate ampicillin resistance. Each vector holds a multiplex tRNA:crRNA cassette to facilitate the cloning of corresponding crRNA via BbsI digestion. The cassette is driven by the ubiquitous *Drosophila* U6:3 promoter (dU6:3) and is transcribed as a single transcript. Upon tRNA maturation, crRNA will be released and ready to form a complex with Cas13 nuclease.

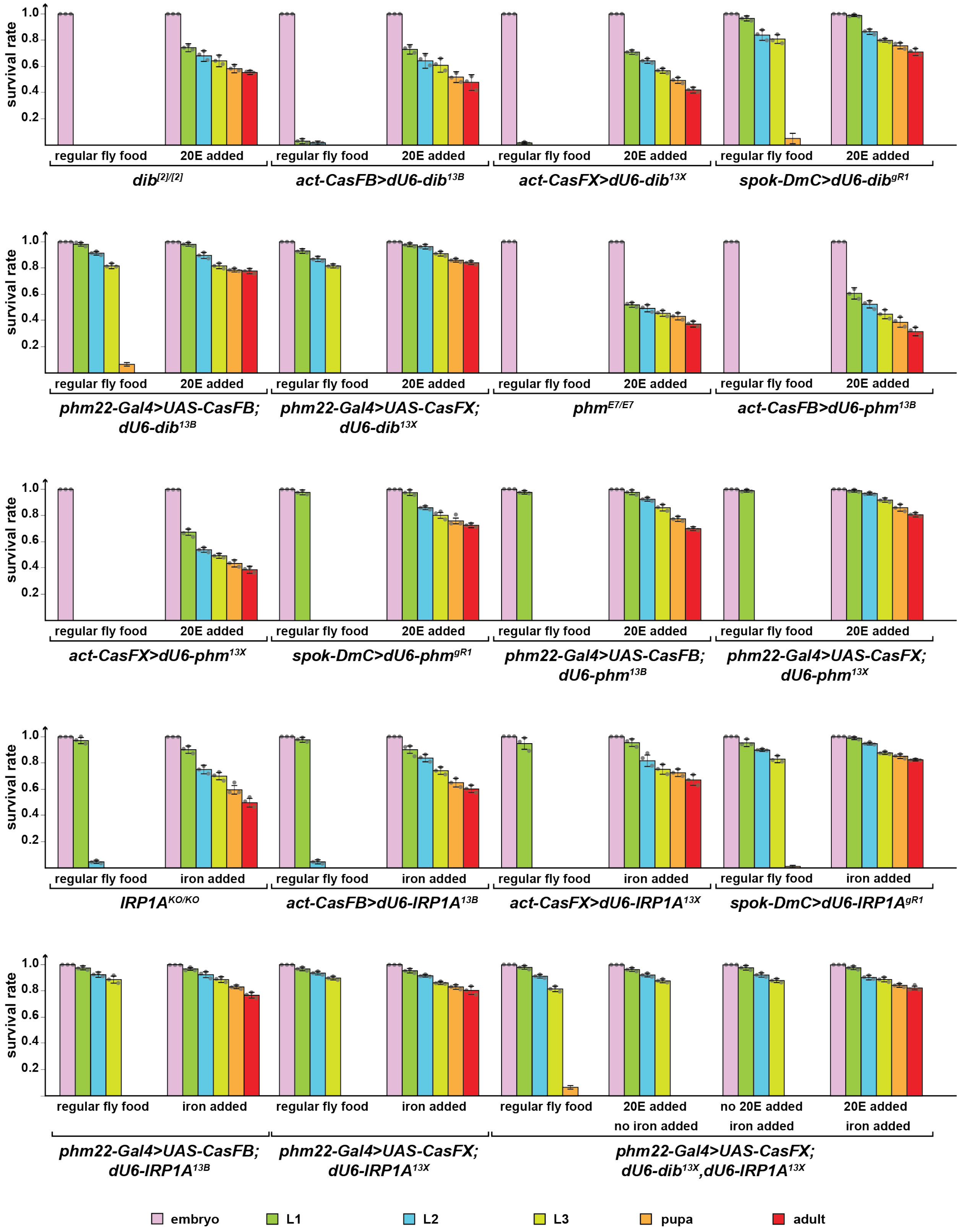

survival rate
1.0
0.8
0.6
0.4
0.2
regular fly food
20E added
iron added
no 20E added
no iron added
dib[2]/[2]
act-CasFB>dU6-dib13B
act-CasFX>dU6-dib13X
spok-DmC>dU6-dibgR1
phm22-Gal4>UAS-CasFB; dU6-dib13B
phm22-Gal4>UAS-CasFX; dU6-dib13X
phmE7/E7
act-CasFB>dU6-phm13B
act-CasFX>dU6-phm13X
spok-DmC>dU6-phmgR1
phm22-Gal4>UAS-CasFB; dU6-phm13B
phm22-Gal4>UAS-CasFX; dU6-phm13X
IRP1AKO/KO
act-CasFB>dU6-IRP1A13B
act-CasFX>dU6-IRP1A13X
spok-DmC>dU6-IRP1AgR1
phm22-Gal4>UAS-CasFB; dU6-IRP1A13B
phm22-Gal4>UAS-CasFX; dU6-IRP1A13X
phm22-Gal4>UAS-CasFX; dU6-dib13X,dU6-IRP1A13X
embryo
L1
L2
L3
pupa
adult

Fig S7: *In vivo* efficiency of *Drosophila* codon-optimized CRISPR/Cas13 variants. Survival rates of classic mutants (*disembodied: dib*^{2}*, phantom: phm*^{E7}, *IRP1A*: *IRP1A*^{KO}), reared on either regular fly food, fly food supplemented with 20-Hydroxyecdysone (20E), or fly food supplemented with iron. Expression of transgenes was either driven by Gal4 (*phm22-Gal4* for prothoracic gland-specific expression) or by direct regulation by an enhancer (*act-CasFX*; *act-CasFB* for ubiquitous expression and *spok-DmC* for prothoracic gland-specific expression). CasFX and CasFB are Cas13 variants from this study, while *spok-DmC* drives the expression of CRISPR/Cas9 [8]. *dU6-dib*^{gR1}, *dU6-phm*^{gR1} and *dU6-IRP1A*^{gR1} are ubiquitously expressed sgRNAs used for CRISPR/Cas9-mediated gene disruption, while all other *dU6* transgenes express crRNAs that work in conjunction with Cas13. Data were normalized to the number of embryos in the starting population. Error bars represent standard deviation.

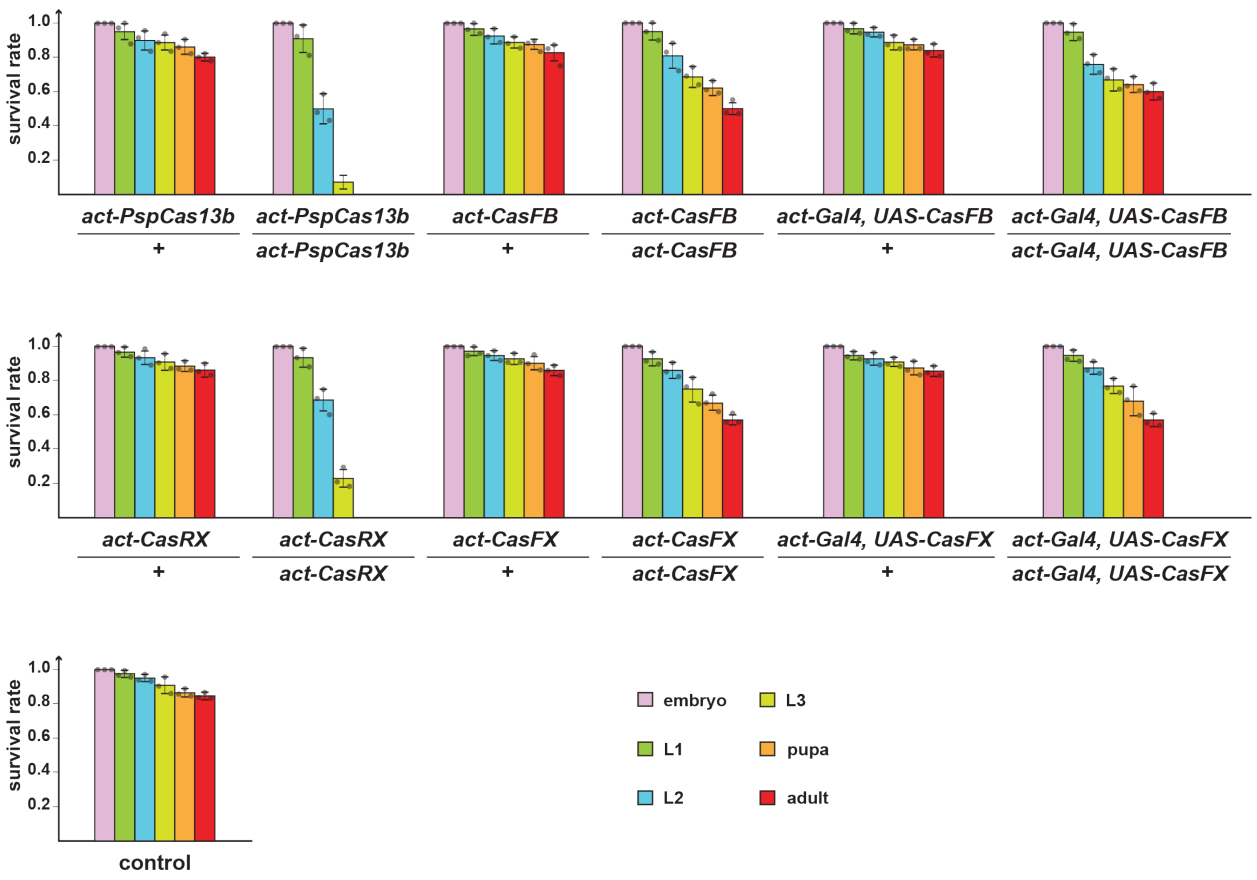

Fig S8: Survival rates of transgenic *Drosophila* lines carrying codon-optimized CRISPR/Cas13. Survival rates of populations heterozygous or homozygous for Cas13 transgenes, including *act-PspCas13b*, *act-CasFB*, *act-Gal4>UAS-CasFB*, *act-CasRX*, *act-CasFX*, and *act-Gal4>UAS-CasFX*. Survival rates of the w^{1118} strain were used as a control. Data were normalized to the number of embryos used in the starting population. Error bars represent standard deviation.
