# Supplementary material for: A versatile toolkit for CRISPR-Cas13-based RNA manipulation in Drosophila
Source: Genome Biol. 2020 Nov 17;21:279. doi: 10.1186/s13059-020-02193-y (PMC7670108; doi:10.1186/s13059-020-02193-y)
Supplement: Supplementary file 3 — Additional file 3. Table S2. List of plasmids. [file 13059_2020_2193_MOESM3_ESM.rtf]

Table S2: List of plasmids
Name	Expression system	Characteristics	
pAc5-CD	Cell culture	Modified from pAFW (DGRC #1111). Expresses dual-reporters eCFP and DsRed under distinct ac5 promoter, also carries NeoR gene and attB integration site.	
pAc5-cD	Cell culture	Modified from pAFW (DGRC #1111). Expresses dual-reporters eCFP* and DsRed under distinct ac5 promoter. eCFP* is eCFP mutant with early stop codon W57*, also carries NeoR gene and attB integration site.	
pAc5-PhiC31	Cell culture	Modified from pAFW (DGRC #1111). Expresses PhiC31 under ac5 promoter for integration reaction, also carries NeoR gene.	
pC13gR01 collection	
pLwaC13a1cr1_1	Cell culture	Expresses Leptotrichia wadei Cas13a (LwaCas13a) as well as crRNA against eCFP at target site 1.	
pCasFA1cr1_1	Cell culture	Expresses CasFA1 as well as crRNA against eCFP at target site 1.	
pCasFA2cr1_1	Cell culture	Expresses CasFA2 as well as crRNA against eCFP at target site 1.	
pCasFA3cr1_1	Cell culture	Expresses CasFA3 as well as crRNA against eCFP at target site 1.	
pCasFA4cr1_1	Cell culture	Expresses CasFA4 as well as crRNA against eCFP at target site 1.	
pCasFA5cr1_1	Cell culture	Expresses CasFA5 as well as crRNA against eCFP at target site 1.	
pCasFA6cr1_1	Cell culture	Expresses CasFA6 as well as crRNA against eCFP at target site 1.	
pCasFA7cr1_1	Cell culture	Expresses CasFA7 as well as crRNA against eCFP at target site 1.	
pCasFA8cr1_1	Cell culture	Expresses CasFA8 as well as crRNA against eCFP at target site 1.	
pCasFA9cr1_1	Cell culture	Expresses CasFA9 as well as crRNA against eCFP at target site 1.	
pCasFA10cr1_1	Cell culture	Expresses CasFA10 as well as crRNA against eCFP at target site 1.	
pPspC13bcr1_1	Cell culture	Expresses Prevotella sp. P25-125 Cas13b (PspCas13b) as well as crRNA against eCFP at target site 1.	
pCasFB1cr1_1	Cell culture	Expresses CasFB1 as well as crRNA against eCFP at target site 1.	
pCasFB2cr1_1	Cell culture	Expresses CasFB2 as well as crRNA against eCFP at target site 1.	
pCasFB3cr1_1	Cell culture	Expresses CasFB3 as well as crRNA against eCFP at target site 1.	
pCasFB4cr1_1	Cell culture	Expresses CasFB4 as well as crRNA against eCFP at target site 1.	
pCasFB5cr1_1	Cell culture	Expresses CasFB5 as well as crRNA against eCFP at target site 1.	
pCasFB6cr1_1	Cell culture	Expresses CasFB6 as well as crRNA against eCFP at target site 1.	
pCasFB7cr1_1	Cell culture	Expresses CasFB7 as well as crRNA against eCFP at target site 1.	
pCasFB8cr1_1	Cell culture	Expresses CasFB8 as well as crRNA against eCFP at target site 1.	
pCasFB9cr1_1	Cell culture	Expresses CasFB9 as well as crRNA against eCFP at target site 1.	
pCasFB10cr1_1	Cell culture	Expresses CasFB10 as well as crRNA against eCFP at target site 1.	
pFneC13ccr1_1	Cell culture	Expresses Fusobacterium perfoetens Cas13c (FneCas13c) as well as crRNA against eCFP at target site 1.	
pCasFC1cr1_1	Cell culture	Expresses CasFC1 as well as crRNA against eCFP at target site 1.	
pCasFC2cr1_1	Cell culture	Expresses CasFC2 as well as crRNA against eCFP at target site 1.	
pCasFC3cr1_1	Cell culture	Expresses CasFC3 as well as crRNA against eCFP at target site 1.	
pCasFC4cr1_1	Cell culture	Expresses CasFC4 as well as crRNA against eCFP at target site 1.	
pCasFC5cr1_1	Cell culture	Expresses CasFC5 as well as crRNA against eCFP at target site 1.	
pCasFC6cr1_1	Cell culture	Expresses CasFC6 as well as crRNA against eCFP at target site 1.	
pCasFC7cr1_1	Cell culture	Expresses CasFC7 as well as crRNA against eCFP at target site 1.	
pCasFC8cr1_1	Cell culture	Expresses CasFC8 as well as crRNA against eCFP at target site 1.	
pCasFC9cr1_1	Cell culture	Expresses CasFC9 as well as crRNA against eCFP at target site 1.	
pCasFC10cr1_1	Cell culture	Expresses CasFC10 as well as crRNA against eCFP at target site 1.	
pCasRXcr1_1	Cell culture	Expresses Ruminococcus flavefaciens XPD3002 Cas13d (CasRX) as well as crRNA against eCFP at target site 1.	
pCasFX1cr1_1	Cell culture	Expresses CasFX1 as well as crRNA against eCFP at target site 1.	
pCasFX2cr1_1	Cell culture	Expresses CasFX2 as well as crRNA against eCFP at target site 1.	
pCasFX3cr1_1	Cell culture	Expresses CasFX3 as well as crRNA against eCFP at target site 1.	
pCasFX4cr1_1	Cell culture	Expresses CasFX4 as well as crRNA against eCFP at target site 1.	
pCasFX5cr1_1	Cell culture	Expresses CasFX5 as well as crRNA against eCFP at target site 1.	
pCasFX6cr1_1	Cell culture	Expresses CasFX6 as well as crRNA against eCFP at target site 1.	
pCasFX7cr1_1	Cell culture	Expresses CasFX7 as well as crRNA against eCFP at target site 1.	
pCasFX8cr1_1	Cell culture	Expresses CasFX8 as well as crRNA against eCFP at target site 1.	
pCasFX9cr1_1	Cell culture	Expresses CasFX9 as well as crRNA against eCFP at target site 1.	
pCasFX10cr1_1	Cell culture	Expresses CasFX10 as well as crRNA against eCFP at target site 1.	
pLwaC13a1cr1_2	Cell culture	Expresses Leptotrichia wadei Cas13a (LwaCas13a) as well as crRNA against eCFP at target site 2.	
pCasFA1cr1_2	Cell culture	Expresses CasFA1 as well as crRNA against eCFP at target site 2.	
pCasFA2cr1_2	Cell culture	Expresses CasFA2 as well as crRNA against eCFP at target site 2.	
pCasFA3cr1_2	Cell culture	Expresses CasFA3 as well as crRNA against eCFP at target site 2.	
pCasFA4cr1_2	Cell culture	Expresses CasFA4 as well as crRNA against eCFP at target site 2.	
pCasFA5cr1_2	Cell culture	Expresses CasFA5 as well as crRNA against eCFP at target site 2.	
pCasFA6cr1_2	Cell culture	Expresses CasFA6 as well as crRNA against eCFP at target site 2.	
pCasFA7cr1_2	Cell culture	Expresses CasFA7 as well as crRNA against eCFP at target site 2.	
pCasFA8cr1_2	Cell culture	Expresses CasFA8 as well as crRNA against eCFP at target site 2.	
pCasFA9cr1_2	Cell culture	Expresses CasFA9 as well as crRNA against eCFP at target site 2.	
pCasFA10cr1_2	Cell culture	Expresses CasFA10 as well as crRNA against eCFP at target site 2.	
pPspC13bcr1_1	Cell culture	Expresses Prevotella sp. P25-125 Cas13b (PspCas13b) as well as crRNA against eCFP at target site 2.	
pCasFB1cr1_2	Cell culture	Expresses CasFB1 as well as crRNA against eCFP at target site 2.	
pCasFB2cr1_2	Cell culture	Expresses CasFB2 as well as crRNA against eCFP at target site 2.	
pCasFB3cr1_2	Cell culture	Expresses CasFB3 as well as crRNA against eCFP at target site 2.	
pCasFB4cr1_2	Cell culture	Expresses CasFB4 as well as crRNA against eCFP at target site 2.	
pCasFB5cr1_2	Cell culture	Expresses CasFB5 as well as crRNA against eCFP at target site 2.	
pCasFB6cr1_2	Cell culture	Expresses CasFB6 as well as crRNA against eCFP at target site 2.	
pCasFB7cr1_2	Cell culture	Expresses CasFB7 as well as crRNA against eCFP at target site 2.	
pCasFB8cr1_2	Cell culture	Expresses CasFB8 as well as crRNA against eCFP at target site 2.	
pCasFB9cr1_2	Cell culture	Expresses CasFB9 as well as crRNA against eCFP at target site 2.	
pCasFB10cr1_2	Cell culture	Expresses CasFB10 as well as crRNA against eCFP at target site 2.	
pFneC13ccr1_1	Cell culture	Expresses Fusobacterium perfoetens Cas13c (FneCas13c) as well as crRNA against eCFP at target site 2.	
pCasFC1cr1_2	Cell culture	Expresses CasFC1 as well as crRNA against eCFP at target site 2.	
pCasFC2cr1_2	Cell culture	Expresses CasFC2 as well as crRNA against eCFP at target site 2.	
pCasFC3cr1_2	Cell culture	Expresses CasFC3 as well as crRNA against eCFP at target site 2.	
pCasFC4cr1_2	Cell culture	Expresses CasFC4 as well as crRNA against eCFP at target site 2.	
pCasFC5cr1_2	Cell culture	Expresses CasFC5 as well as crRNA against eCFP at target site 2.	
pCasFC6cr1_2	Cell culture	Expresses CasFC6 as well as crRNA against eCFP at target site 2.	
pCasFC7cr1_2	Cell culture	Expresses CasFC7 as well as crRNA against eCFP at target site 2.	
pCasFC8cr1_2	Cell culture	Expresses CasFC8 as well as crRNA against eCFP at target site 2.	
pCasFC9cr1_2	Cell culture	Expresses CasFC9 as well as crRNA against eCFP at target site 2.	
pCasFC10cr1_2	Cell culture	Expresses CasFC10 as well as crRNA against eCFP at target site 2.	
pCasRXcr1_2	Cell culture	Expresses Ruminococcus flavefaciens XPD3002 Cas13d (CasRX) as well as crRNA against eCFP at target site 2.	
pCasFX1cr1_2	Cell culture	Expresses CasFX1 as well as crRNA against eCFP at target site 2.	
pCasFX2cr1_2	Cell culture	Expresses CasFX2 as well as crRNA against eCFP at target site 2.	
pCasFX3cr1_2	Cell culture	Expresses CasFX3 as well as crRNA against eCFP at target site 2.	
pCasFX4cr1_2	Cell culture	Expresses CasFX4 as well as crRNA against eCFP at target site 2.	
pCasFX5cr1_2	Cell culture	Expresses CasFX5 as well as crRNA against eCFP at target site 2.	
pCasFX6cr1_2	Cell culture	Expresses CasFX6 as well as crRNA against eCFP at target site 2.	
pCasFX7cr1_2	Cell culture	Expresses CasFX7 as well as crRNA against eCFP at target site 2.	
pCasFX8cr1_2	Cell culture	Expresses CasFX8 as well as crRNA against eCFP at target site 2.	
pCasFX9cr1_2	Cell culture	Expresses CasFX9 as well as crRNA against eCFP at target site 2.	
pCasFX10cr1_2	Cell culture	Expresses CasFX10 as well as crRNA against eCFP at target site 2.	
pLwaC13a1cr1_0	Cell culture	Expresses Leptotrichia wadei Cas13a (LwaCas13a) as well as no-targeting crRNA.	
pCasFA1cr1_0	Cell culture	Expresses CasFA1 as well as no-targeting crRNA.	
pCasFA2cr1_0	Cell culture	Expresses CasFA2 as well as no-targeting crRNA.	
pCasFA3cr1_0	Cell culture	Expresses CasFA3 as well as no-targeting crRNA.	
pCasFA4cr1_0	Cell culture	Expresses CasFA4 as well as no-targeting crRNA.	
pCasFA5cr1_0	Cell culture	Expresses CasFA5 as well as no-targeting crRNA.	
pCasFA6cr1_0	Cell culture	Expresses CasFA6 as well as no-targeting crRNA.	
pCasFA7cr1_0	Cell culture	Expresses CasFA7 as well as no-targeting crRNA.	
pCasFA8cr1_0	Cell culture	Expresses CasFA8 as well as no-targeting crRNA.	
pCasFA9cr1_0	Cell culture	Expresses CasFA9 as well as no-targeting crRNA.	
pCasFA10cr1_0	Cell culture	Expresses CasFA10 as well as no-targeting crRNA.	
pPspC13bcr1_0	Cell culture	Expresses Prevotella sp. P25-125 Cas13b (PspCas13b) as well as no-targeting crRNA.	
pCasFB1cr1_0	Cell culture	Expresses CasFB1 as well as no-targeting crRNA.	
pCasFB2cr1_0	Cell culture	Expresses CasFB2 as well as no-targeting crRNA.	
pCasFB3cr1_0	Cell culture	Expresses CasFB3 as well as no-targeting crRNA.	
pCasFB4cr1_0	Cell culture	Expresses CasFB4 as well as no-targeting crRNA.	
pCasFB5cr1_0	Cell culture	Expresses CasFB5 as well as no-targeting crRNA.	
pCasFB6cr1_0	Cell culture	Expresses CasFB6 as well as no-targeting crRNA.	
pCasFB7cr1_0	Cell culture	Expresses CasFB7 as well as no-targeting crRNA.	
pCasFB8cr1_0	Cell culture	Expresses CasFB8 as well as no-targeting crRNA.	
pCasFB9cr1_0	Cell culture	Expresses CasFB9 as well as no-targeting crRNA.	
pCasFB10cr1_0	Cell culture	Expresses CasFB10 as well as no-targeting crRNA.	
pFpeC13ccr1_0	Cell culture	Expresses Fusobacterium perfoetens Cas13c (FpeCas13c) as well as no-targeting crRNA.	
pCasFC1cr1_0	Cell culture	Expresses CasFC1 as well as no-targeting crRNA.	
pCasFC2cr1_0	Cell culture	Expresses CasFC2 as well as no-targeting crRNA.	
pCasFC3cr1_0	Cell culture	Expresses CasFC3 as well as no-targeting crRNA.	
pCasFC4cr1_0	Cell culture	Expresses CasFC4 as well as no-targeting crRNA.	
pCasFC5cr1_0	Cell culture	Expresses CasFC5 as well as no-targeting crRNA.	
pCasFC6cr1_0	Cell culture	Expresses CasFC6 as well as no-targeting crRNA.	
pCasFC7cr1_0	Cell culture	Expresses CasFC7 as well as no-targeting crRNA.	
pCasFC8cr1_0	Cell culture	Expresses CasFC8 as well as no-targeting crRNA.	
pCasFC9cr1_0	Cell culture	Expresses CasFC9 as well as no-targeting crRNA.	
pCasFC10cr1_0	Cell culture	Expresses CasFC10 as well as no-targeting crRNA.	
pCasRXcr1_0	Cell culture	Expresses Ruminococcus flavefaciens XPD3002 Cas13d (CasRX) as well as no-targeting crRNA.	
pCasFX1cr1_0	Cell culture	Expresses CasFX1 as well as no-targeting crRNA.	
pCasFX2cr1_0	Cell culture	Expresses CasFX2 as well as no-targeting crRNA.	
pCasFX3cr1_0	Cell culture	Expresses CasFX3 as well as no-targeting crRNA.	
pCasFX4cr1_0	Cell culture	Expresses CasFX4 as well as no-targeting crRNA.	
pCasFX5cr1_0	Cell culture	Expresses CasFX5 as well as no-targeting crRNA.	
pCasFX6cr1_0	Cell culture	Expresses CasFX6 as well as no-targeting crRNA.	
pCasFX7cr1_0	Cell culture	Expresses CasFX7 as well as no-targeting crRNA.	
pCasFX8cr1_0	Cell culture	Expresses CasFX8 as well as no-targeting crRNA.	
pCasFX9cr1_0	Cell culture	Expresses CasFX9 as well as no-targeting crRNA.	
pCasFX10cr1_0	Cell culture	Expresses CasFX10 as well as no-targeting crRNA.	
Plasmids for specificity evaluation	
pCasFA5_cr1-a3	Cell culture	Expresses CasFA5 as well as crRNA with single mismatch at the 3rd nucleotide. 	
pCasFA5_cr1-a6	Cell culture	Expresses CasFA5 as well as crRNA with single mismatch at the 6th nucleotide. 	
pCasFA5_cr1-a9	Cell culture	Expresses CasFA5 as well as crRNA with single mismatch at the 9th nucleotide. 	
pCasFA5_cr1-a12	Cell culture	Expresses CasFA5 as well as crRNA with single mismatch at the 12th nucleotide. 	
pCasFA5_cr1-a15	Cell culture	Expresses CasFA5 as well as crRNA with single mismatch at the 15th nucleotide. 	
pCasFA5_cr1-a18	Cell culture	Expresses CasFA5 as well as crRNA with single mismatch at the 18th nucleotide. 	
pCasFA5_cr1-a21	Cell culture	Expresses CasFA5 as well as crRNA with single mismatch at the 21st nucleotide. 	
pCasFA5_cr1-a24	Cell culture	Expresses CasFA5 as well as crRNA with single mismatch at the 24th nucleotide. 	
pCasFA5_cr1-a27	Cell culture	Expresses CasFA5 as well as crRNA with single mismatch at the 27th nucleotide. 	
pCasFA5_cr1-a28	Cell culture	Expresses CasFA5 as well as crRNA with single mismatch at the 28th nucleotide. 	
pCasFC4_cr1-a3	Cell culture	Expresses CasFC4 as well as crRNA with single mismatch at the 3rd nucleotide. 	
pCasFC4_cr1-a6	Cell culture	Expresses CasFC4 as well as crRNA with single mismatch at the 6th nucleotide. 	
pCasFC4_cr1-a9	Cell culture	Expresses CasFC4 as well as crRNA with single mismatch at the 9th nucleotide. 	
pCasFC4_cr1-a12	Cell culture	Expresses CasFC4 as well as crRNA with single mismatch at the 12th nucleotide. 	
pCasFC4_cr1-a15	Cell culture	Expresses CasFC4 as well as crRNA with single mismatch at the 15th nucleotide. 	
pCasFC4_cr1-a18	Cell culture	Expresses CasFC4 as well as crRNA with single mismatch at the 18th nucleotide. 	
pCasFC4_cr1-a21	Cell culture	Expresses CasFC4 as well as crRNA with single mismatch at the 21st nucleotide. 	
pCasFC4_cr1-a24	Cell culture	Expresses CasFC4 as well as crRNA with single mismatch at the 24th nucleotide. 	
pCasFC4_cr1-a27	Cell culture	Expresses CasFC4 as well as crRNA with single mismatch at the 27th nucleotide. 	
pCasFC4_cr1-a30	Cell culture	Expresses CasFC4 as well as crRNA with single mismatch at the 30th nucleotide. 	
pCasFB5_cr1-a3	Cell culture	Expresses CasFB5 as well as crRNA with single mismatch at the 3rd nucleotide. 	
pCasFB5_cr1-a6	Cell culture	Expresses CasFB5 as well as crRNA with single mismatch at the 6th nucleotide. 	
pCasFB5_cr1-a9	Cell culture	Expresses CasFB5 as well as crRNA with single mismatch at the 9th nucleotide. 	
pCasFB5_cr1-a12	Cell culture	Expresses CasFB5 as well as crRNA with single mismatch at the 12th nucleotide. 	
pCasFB5_cr1-a15	Cell culture	Expresses CasFB5 as well as crRNA with single mismatch at the 15th nucleotide. 	
pCasFB5_cr1-a18	Cell culture	Expresses CasFB5 as well as crRNA with single mismatch at the 18th nucleotide. 	
pCasFB5_cr1-a21	Cell culture	Expresses CasFB5 as well as crRNA with single mismatch at the 21st nucleotide. 	
pCasFB5_cr1-a24	Cell culture	Expresses CasFB5 as well as crRNA with single mismatch at the 24th nucleotide. 	
pCasFB5_cr1-a27	Cell culture	Expresses CasFB5 as well as crRNA with single mismatch at the 27th nucleotide. 	
pCasFB5_cr1-a30	Cell culture	Expresses CasFB5 as well as crRNA with single mismatch at the 30th nucleotide. 	
pCasFB8_cr1-a3	Cell culture	Expresses CasFB8 as well as crRNA with single mismatch at the 3rd nucleotide. 	
pCasFB8_cr1-a6	Cell culture	Expresses CasFB8 as well as crRNA with single mismatch at the 6th nucleotide. 	
pCasFB8_cr1-a9	Cell culture	Expresses CasFB8 as well as crRNA with single mismatch at the 9th nucleotide. 	
pCasFB8_cr1-a12	Cell culture	Expresses CasFB8 as well as crRNA with single mismatch at the 12th nucleotide. 	
pCasFB8_cr1-a15	Cell culture	Expresses CasFB8 as well as crRNA with single mismatch at the 15th nucleotide. 	
pCasFB8_cr1-a18	Cell culture	Expresses CasFB8 as well as crRNA with single mismatch at the 18th nucleotide. 	
pCasFB8_cr1-a21	Cell culture	Expresses CasFB8 as well as crRNA with single mismatch at the 21st nucleotide. 	
pCasFB8_cr1-a24	Cell culture	Expresses CasFB8 as well as crRNA with single mismatch at the 24th nucleotide. 	
pCasFB8_cr1-a27	Cell culture	Expresses CasFB8 as well as crRNA with single mismatch at the 27th nucleotide. 	
pCasFB8_cr1-a30	Cell culture	Expresses CasFB8 as well as crRNA with single mismatch at the 30th nucleotide. 	
pCasFX4_cr1-a1	Cell culture	Expresses CasFX4 as well as crRNA with single mismatch at the 1st nucleotide. 	
pCasFX4_cr1-a3	Cell culture	Expresses CasFX4 as well as crRNA with single mismatch at the 3rd nucleotide. 	
pCasFX4_cr1-a6	Cell culture	Expresses CasFX4 as well as crRNA with single mismatch at the 6th nucleotide. 	
pCasFX4_cr1-a9	Cell culture	Expresses CasFX4 as well as crRNA with single mismatch at the 9th nucleotide. 	
pCasFX4_cr1-a12	Cell culture	Expresses CasFX4 as well as crRNA with single mismatch at the 12th nucleotide. 	
pCasFX4_cr1-a15	Cell culture	Expresses CasFX4 as well as crRNA with single mismatch at the 15th nucleotide. 	
pCasFX4_cr1-a18	Cell culture	Expresses CasFX4 as well as crRNA with single mismatch at the 18th nucleotide. 	
pCasFX4_cr1-a21	Cell culture	Expresses CasFX4 as well as crRNA with single mismatch at the 21st nucleotide. 	
pCasFX4_cr1-a24	Cell culture	Expresses CasFX4 as well as crRNA with single mismatch at the 24th nucleotide. 	
pCasFX4_cr1-a27	Cell culture	Expresses CasFX4 as well as crRNA with single mismatch at the 27th nucleotide. 	
pCasFX4_cr1-a30	Cell culture	Expresses CasFX4 as well as crRNA with single mismatch at the 30th nucleotide. 	
pCasFX_cr1-a1	Cell culture	Expresses CasFX8 as well as crRNA with single mismatch at the 1st nucleotide.	
pCasFX8_cr1-a3	Cell culture	Expresses CasFX8 as well as crRNA with single mismatch at the 3rd nucleotide. 	
pCasFX8_cr1-a6	Cell culture	Expresses CasFX8 as well as crRNA with single mismatch at the 6th nucleotide. 	
pCasFX8_cr1-a9	Cell culture	Expresses CasFX8 as well as crRNA with single mismatch at the 9th nucleotide. 	
pCasFX8_cr1-a12	Cell culture	Expresses CasFX8 as well as crRNA with single mismatch at the 12th nucleotide. 	
pCasFX8_cr1-a15	Cell culture	Expresses CasFX8 as well as crRNA with single mismatch at the 15th nucleotide. 	
pCasFX8_cr1-a18	Cell culture	Expresses CasFX8 as well as crRNA with single mismatch at the 18th nucleotide. 	
pCasFX8_cr1-a21	Cell culture	Expresses CasFX8 as well as crRNA with single mismatch at the 21st nucleotide. 	
pCasFX8_cr1-a24	Cell culture	Expresses CasFX8 as well as crRNA with single mismatch at the 24th nucleotide. 	
pCasFX8_cr1-a27	Cell culture	Expresses CasFX8 as well as crRNA with single mismatch at the 27th nucleotide. 	
pCasFX8_cr1-a30	Cell culture	Expresses CasFX8 as well as crRNA with single mismatch at the 30th nucleotide. 	
pCasFA5_cr1-a3+6	Cell culture	Expresses CasFA5 as well as crRNA with dual mismatches at the 3rd and 6th nucleotides. 	
pCasFA5_cr1-a3+15	Cell culture	Expresses CasFA5 as well as crRNA with dual mismatches at the 3rd and 15th nucleotides. 	
pCasFA5_cr1-a3+28	Cell culture	Expresses CasFA5 as well as crRNA with dual mismatches at the 3rd and 28th nucleotides. 	
pCasFA5_cr1-a3+6+15	Cell culture	Expresses CasFA5 as well as crRNA with triple mismatches at the 3rd, 6th and 15th nucleotides. 	
pCasFA5_cr1-a3+6+28	Cell culture	Expresses CasFA5 as well as crRNA with triple mismatches at the 3rd, 6th and 28th nucleotides. 	
pCasFA5_cr1-a3+6+15+28	Cell culture	Expresses CasFA5 as well as crRNA with quadruple mismatches at the 3rd, 6th, 15th and 28th nucleotides. 	
pCasFC4_cr1-a3+6	Cell culture	Expresses CasFC4 as well as crRNA with dual mismatches at the 3rd and 6th nucleotides. 	
pCasFC4_cr1-a3+15	Cell culture	Expresses CasFC4 as well as crRNA with dual mismatches at the 3rd and 15th nucleotides. 	
pCasFC4_cr1-a3+30	Cell culture	Expresses CasFC4 as well as crRNA with dual mismatches at the 3rd and 30th nucleotides. 	
pCasFC4_cr1-a3+6+15	Cell culture	Expresses CasFC4 as well as crRNA with triple mismatches at the 3rd, 6th and 15th nucleotides. 	
pCasFC4_cr1-a3+6+30	Cell culture	Expresses CasFC4 as well as crRNA with triple mismatches at the 3rd, 6th and 30th nucleotides. 	
pCasFC4_cr1-a3+6+15+30	Cell culture	Expresses CasFC4 as well as crRNA with quadruple mismatches at the 3rd, 6th, 15th and 30th nucleotides. 	
pCasFB5_cr1-a3+6	Cell culture	Expresses CasFB5 as well as crRNA with dual mismatches at the 3rd and 6th nucleotides. 	
pCasFB5_cr1-a3+15	Cell culture	Expresses CasFB5 as well as crRNA with dual mismatches at the 3rd and 15th nucleotides. 	
pCasFB5_cr1-a3+30	Cell culture	Expresses CasFB5 as well as crRNA with dual mismatches at the 3rd and 30th nucleotides. 	
pCasFB5_cr1-a3+6+15	Cell culture	Expresses CasFB5 as well as crRNA with triple mismatches at the 3rd, 6th and 15th nucleotides. 	
pCasFB5_cr1-a3+6+30	Cell culture	Expresses CasFB5 as well as crRNA with triple mismatches at the 3rd, 6th and 30th nucleotides. 	
pCasFB5_cr1-a3+6+15+30	Cell culture	Expresses CasFB5 as well as crRNA with quadruple mismatches at the 3rd, 6th, 15th and 30th nucleotides. 	
pCasFB8_cr1-a3+6	Cell culture	Expresses CasFB8 as well as crRNA with dual mismatches at the 3rd and 6th nucleotides. 	
pCasFB8_cr1-a3+15	Cell culture	Expresses CasFB8 as well as crRNA with dual mismatches at the 3rd and 15th nucleotides. 	
pCasFB8_cr1-a3+30	Cell culture	Expresses CasFB8 as well as crRNA with dual mismatches at the 3rd and 30th nucleotides. 	
pCasFB8_cr1-a3+6+15	Cell culture	Expresses CasFB8 as well as crRNA with triple mismatches at the 3rd, 6th and 15th nucleotides. 	
pCasFB8_cr1-a3+6+30	Cell culture	Expresses CasFB8 as well as crRNA with triple mismatches at the 3rd, 6th and 30th nucleotides. 	
pCasFB8_cr1-a3+6+15+30	Cell culture	Expresses CasFB8 as well as crRNA with quadruple mismatches at the 3rd, 6th, 15th and 30th nucleotides. 	
pCasFX4_cr1-a3+6	Cell culture	Expresses CasFX4 as well as crRNA with dual mismatches at the 3rd and 6th nucleotides. 	
pCasFX4_cr1-a3+15	Cell culture	Expresses CasFX4 as well as crRNA with dual mismatches at the 3rd and 15th nucleotides. 	
pCasFX4_cr1-a3+30	Cell culture	Expresses CasFX4 as well as crRNA with dual mismatches at the 3rd and 30th nucleotides. 	
pCasFX4_cr1-a3+6+15	Cell culture	Expresses CasFX4 as well as crRNA with triple mismatches at the 3rd, 6th and 15th nucleotides. 	
pCasFX4_cr1-a3+6+30	Cell culture	Expresses CasFX4 as well as crRNA with triple mismatches at the 3rd, 6th and 30th nucleotides. 	
pCasFX4_cr1-a3+6+15+30	Cell culture	Expresses CasFX4 as well as crRNA with quadruple mismatches at the 3rd, 6th, 15th and 30th nucleotides. 	
pCasFX8_cr1-a3+6	Cell culture	Expresses CasFX8 as well as crRNA with dual mismatches at the 3rd and 6th nucleotides. 	
pCasFX8_cr1-a3+15	Cell culture	Expresses CasFX8 as well as crRNA with dual mismatches at the 3rd and 15th nucleotides. 	
pCasFX8_cr1-a3+30	Cell culture	Expresses CasFX8 as well as crRNA with dual mismatches at the 3rd and 30th nucleotides. 	
pCasFX8_cr1-a3+6+15	Cell culture	Expresses CasFX8 as well as crRNA with triple mismatches at the 3rd, 6th and 15th nucleotides. 	
pCasFX8_cr1-a3+6+30	Cell culture	Expresses CasFX8 as well as crRNA with triple mismatches at the 3rd, 6th and 30th nucleotides. 	
pCasFX8_cr1-a3+6+15+30	Cell culture	Expresses CasFX8 as well as crRNA with quadruple mismatches at the 3rd, 6th, 15th and 30th nucleotides. 	
Plasmids for nuclease dead CasFX	
pdCasFX_cr1	Cell culture	Expresses nuclease-dead CasFX_04 (dCasFX) as well as crRNA against eCFP at target site 1	
pdCasFX_cr1C	Cell culture	Expresses dCasFX as well as crRNA cassette with no target site.	
Plasmids for transcript tracking	
pdCasFX_crA1	Cell culture	Expresses dCasFX as well as crRNA targeting site 300 bp upstream of IRE on Fer1HCH RA mRNA.	
pdCasFX_crA2	Cell culture	Expresses dCasFX as well as crRNA targeting site 150 bp upstream of IRE on Fer1HCH RA mRNA.	
pdCasFX_crA3	Cell culture	Expresses dCasFX as well as crRNA targeting IRE on Fer1HCH RA mRNA.	
pdCasFX_crA4	Cell culture	Expresses dCasFX as well as crRNA targeting site 150 bp downstream of IRE on Fer1HCH RA.	
pdCasFX_crA5	Cell culture	Expresses dCasFX as well as crRNA targeting site 300bp downstream of IRE on Fer1HCH RA.	
pdCasFX_crA6	Cell culture	Expresses dCasFX as well as crRNA targeting site 450bp downstream of IRE on Fer1HCH RA.	
pdCasFX_crA7	Cell culture	Expresses dCasFX as well as crRNA targeting site 600bp downstream of IRE on Fer1HCH RA.	
pdCasFX_crA8	Cell culture	Expresses dCasFX as well as crRNA targeting site 750bp downstream of IRE on Fer1HCH RA.	
pdCasFX_crA9	Cell culture	Expresses dCasFX as well as crRNA targeting site 900 bp downstream of IRE on Fer1HCH RA.	
pdCasFX_crAC	Cell culture	Expresses dCasFX as well as crRNA cassette with no target site.	
pAFW-IRP1AC450S-Fer1HCH RA	Cell culture	Expresses 3xFlag-tagged IRP1AC450S as well as Fer1HCH RA.	
Plasmids for mitochondrial-encoded RNA target	
pCasFXMT_cr1C	Cell culture	Expresses CasFX fused with mitochondrial signaling sequence (CasFXMT) and crRNA cassette with no target site.	
pCasFXMT_cr1COXI-1	Cell culture	Expresses CasFXMT and crRNA1 targeting COXI transcript	
pCasFXMT_cr1COXI-2	Cell culture	Expresses CasFXMT and crRNA2 targeting COXI transcript	
pCasFXMT_cr1COXI-3	Cell culture	Expresses CasFXMT and crRNA3 targeting COXI transcript	
pCasFXMT_cr1COXI-4	Cell culture	Expresses CasFXMT and crRNA4 targeting COXI transcript	
pCasFXMT_cr1COXII-1	Cell culture	Expresses CasFXMT and crRNA1 targeting COXII transcript	
pCasFXMT_cr1COXII-2	Cell culture	Expresses CasFXMT and crRNA2 targeting COXII transcript	
pCasFXMT_cr1COXII-3	Cell culture	Expresses CasFXMT and crRNA3 targeting COXII transcript	
pCasFXMT_cr1COXII-3	Cell culture	Expresses CasFXMT and crRNA3 targeting COXII transcript	
Plasmids for RNA editing application	
pFREPAIRv2-cr50*2	Cell culture	Expresses the dCasFX fused with ADAR2DD (FREPAIRv2) together with crRNA carrying 50-nt long spacer and a mismatch at location 2 from the first nucleotide against W57* on eCFP* in Sg4-cD cell line.	
pFREPAIRv2-cr50*10	Cell culture	Expresses the dCasFX fused with ADAR2DD (FREPAIRv2) together with crRNA carrying 50-nt long spacer and a mismatch at location 10 from the first nucleotide against W57* on eCFP* in Sg4-cD cell line.	
pFREPAIRv2-cr50*18	Cell culture	Expresses the dCasFX fused with ADAR2DD (FREPAIRv2) together with crRNA carrying 50-nt long spacer and a mismatch at location 18 from the first nucleotide against W57* on eCFP* in Sg4-cD cell line.	
pFREPAIRv2-cr50*26	Cell culture	Expresses the dCasFX fused with ADAR2DD (FREPAIRv2) together with crRNA carrying 50-nt long spacer and a mismatch at location 26 from the first nucleotide against W57* on eCFP* in Sg4-cD cell line.	
pFREPAIRv2-cr50*34	Cell culture	Expresses the dCasFX fused with ADAR2DD (FREPAIRv2) together with crRNA carrying 50-nt long spacer and a mismatch at location 34 from the first nucleotide against W57* on eCFP* in Sg4-cD cell line.	
pFREPAIRv2-cr50*42	Cell culture	Expresses the dCasFX fused with ADAR2DD (FREPAIRv2) together with crRNA carrying 50-nt long spacer and a mismatch at location 42 from the first nucleotide against W57* on eCFP* in Sg4-cD cell line.	
pFREPAIRv50-cr*50	Cell culture	Expresses the dCasFX fused with ADAR2DD (FREPAIRv2) together with crRNA carrying 50-nt long spacer and a mismatch at location 50 from the first nucleotide against W57* on eCFP* in Sg4-cD cell line.	
pFREPAIRv2-cr30*26	Cell culture	Expresses the dCasFX fused with ADAR2DD (FREPAIRv2) together with crRNA carrying 30-nt long spacer and a mismatch at location 26 from the first nucleotide against W57* on eCFP* in Sg4-cD cell line.	
pFREPAIRv2-cr40*26	Cell culture	Expresses the dCasFX fused with ADAR2DD (FREPAIRv2) together with crRNA carrying 40-nt long spacer and a mismatch at location 26 from the first nucleotide against W57* on eCFP* in Sg4-cD cell line.	
pFREPAIRv2-cr60*26	Cell culture	Expresses the dCasFX fused with ADAR2DD (FREPAIRv2) together with crRNA carrying 60-nt long spacer and a mismatch at location 26 from the first nucleotide against W57* on eCFP* in Sg4-cD cell line.	
pFREPAIRv2-cr70*26	Cell culture	Expresses the dCasFX fused with ADAR2DD (FREPAIRv2) together with crRNA carrying 70-nt long spacer and a mismatch at location 26 from the first nucleotide against W57* on eCFP* in Sg4-cD cell line.	
pFREPAIRv2-cr80*26	Cell culture	Expresses the dCasFX fused with ADAR2DD (FREPAIRv2) together with crRNA carrying 80-nt long spacer and a mismatch at location 26 from the first nucleotide against W57* on eCFP* in Sg4-cD cell line.	
General gateway-Cas13 variants plasmids for transgenic fly	
CasFB	Transgenic fly	Expresses the 3xHA tagged CasFB5 for RNA cleavage, carries embedded attB integration site.	
CasFX	Transgenic fly	Expresses the 3xHA tagged CasFX4 for RNA cleavage, carries embedded attB integration site.	
pC13X	Transgenic fly	Expresses the CasFX-compatible crRNA under control of dU6:3 promoter, carries embedded attB integration site.	
pC13B	Transgenic fly	Expresses the CasFB-compatible crRNA under control of dU6:3 promoter, carries embedded attB integration site.	
