# Supplementary material for: A versatile toolkit for CRISPR-Cas13-based RNA manipulation in Drosophila
Source: Genome Biol. 2020 Nov 17;21:279. doi: 10.1186/s13059-020-02193-y (PMC7670108; doi:10.1186/s13059-020-02193-y)
Supplement: Supplementary file 4 — Additional file 4. Table S3. Primers. [file 13059_2020_2193_MOESM4_ESM.rtf]

Table S3: Primers	
Primer name	Primer sequences (5' - 3')	
pC13acr01	
attB1 Lwa FP	TCAAGTTTGTACAAAAAAGCAGGCTATGAAAGTGACCAAGGTCGAC	
attB2 Lwa RP	CCCACTTTGTACAAGAAAGCTGGGTTTATTCCAGGGCCTTGTACTCGAACATGAC	
attB1 CasFA1 FP	TCAAGTTTGTACAAAAAAGCAGGCTATGAAGGTGACCAAGGTGGATGGCATC	
attB2 CasFA1 RP	CCCACTTTGTACAAGAAAGCTGGGTTTACTCCAGGGCCTTGTACTCGAACATC	
attB1 CasFA2 FP	TCAAGTTTGTACAAAAAAGCAGGCTATGAAGGTGACCAAGGTCGACGGCATC	
attB2 CasFA2 RP	CCCACTTTGTACAAGAAAGCTGGGTTTATTCCAGGGCCTTGTACTCGAACATGAC	
attB1 CasFA3 FP	TCAAGTTTGTACAAAAAAGCAGGCTATGAAAGTGACCAAGGTCGACGGCATCAG	
attB2 CasFA3 RP	CCCACTTTGTACAAGAAAGCTGGGTTTACTCCAGGGCCTTGTACTCGAACATGAC	
attB1 CasFA4 FP	TCAAGTTTGTACAAAAAAGCAGGCTATGAAGGTGACCAAGGTCGACGGCATCAG	
attB2 CasFA4 RP	CCCACTTTGTACAAGAAAGCTGGGTTTACTCCAGGGCCTTGTACTCGAACATGACCTTC	
attB1 CasFA5 FP	TCAAGTTTGTACAAAAAAGCAGGCTATGAAGGTGACCAAGGTCGACGGCATCAGCCAC	
attB2 CasFA5 RP	CCCACTTTGTACAAGAAAGCTGGGTTTACTCCAGGGCCTTGTACTCGAACATGAC	
attB1 CasFA6 FP	TCAAGTTTGTACAAAAAAGCAGGCTATGAAGGTGACCAAGGTCGACGGCATC	
attB2 CasFA6 RP	CCCACTTTGTACAAGAAAGCTGGGTTTACTCCAGGGCCTTGTACTCGAACATGACCTTCAC	
attB1 CasFA7 FP	TCAAGTTTGTACAAAAAAGCAGGCTATGAAGGTGACCAAGGTCGACGGCATCAGCCAC	
attB2 CasFA7 RP	CCCACTTTGTACAAGAAAGCTGGGTTTACTCCAGGGCCTTGTACTCGAACATGACCTTC	
attB1 CasFA8 FP	TCAAGTTTGTACAAAAAAGCAGGCTATGAAGGTGACCAAGGTCGACGGCATC	
attB2 CasFA8 RP	CCCACTTTGTACAAGAAAGCTGGGTTTACTCCAGGGCCTTGTACTCGAACATGACCTTC	
attB1 CasFA9 FP	TCAAGTTTGTACAAAAAAGCAGGCTATGAAGGTGACCAAGGTCGACGGCATCAGCCAC	
attB2 CasFA9 RP	CCCACTTTGTACAAGAAAGCTGGGTTTACTCCAGGGCCTTGTACTCGAACATGACCTTC	
attB1 CasFA10 FP	TCAAGTTTGTACAAAAAAGCAGGCTATGAAGGTGACCAAGGTCGACGGCATCAGCCAC	
attB2 CasFA10 RP	CCCACTTTGTACAAGAAAGCTGGGTTTACTCCAGGGCCTTGTACTCGAACATGACCTTC	
pC13bcr01	
attB1 Psp FP	TCAAGTTTGTACAAAAAAGCAGGCTATGAACATCCCCGCTCTGGTGGAAAAC	
attB2 Psp RP	CCCACTTTGTACAAGAAAGCTGGGTTTACTTCATGATGGCGTACTCCCCAAAG	
attB1 CasFB1 FP	TCAAGTTTGTACAAAAAAGCAGGCTATGAACATCCCCGCCCTGGTGGAGAAC	
attB2 CasFB1 RP	CCCACTTTGTACAAGAAAGCTGGGTTTACTTCATGATGGCGTACTCGCCGAAG	
attB1 CasFB2 FP	TCAAGTTTGTACAAAAAAGCAGGCTATGAACATCCCCGCTCTGGTGGAAAAC	
attB2 CasFB2 RP	CCCACTTTGTACAAGAAAGCTGGGTTTACTTCATGATGGCGTACTCGCCAAAG	
attB1 CasFB3 FP	TCAAGTTTGTACAAAAAAGCAGGCTATGAACATCCCCGCTCTGGTGGAGAAC	
attB2 CasFB3 RP	CCCACTTTGTACAAGAAAGCTGGGTTTACTTCATGATGGCGTACTCGCCAAAG	
attB1 CasFB4 FP	TCAAGTTTGTACAAAAAAGCAGGCTATGAACATCCCCGCTCTGGTGGAGAAC	
attB2 CasFB4 RP	CCCACTTTGTACAAGAAAGCTGGGTTTACTTCATGATGGCGTACTCGCCAAAG	
attB1 CasFB5 FP	TCAAGTTTGTACAAAAAAGCAGGCTATGAACATCCCCGCTCTGGTGGAGAAC	
attB2 CasFB5 RP	CCCACTTTGTACAAGAAAGCTGGGTTTACTTCATGATGGCGTACTCGCCAAAG	
attB1 CasFB6 FP	TCAAGTTTGTACAAAAAAGCAGGCTATGAACATCCCCGCTCTGGTGGAGAAC	
attB2 CasFB6 RP	CCCACTTTGTACAAGAAAGCTGGGTTTACTTCATGATGGCGTACTCCCCAAAG	
attB1 CasFB7 FP	TCAAGTTTGTACAAAAAAGCAGGCTATGAACATCCCCGCTCTGGTGGAGAAC	
attB2 CasFB7 RP	CCCACTTTGTACAAGAAAGCTGGGTTTACTTCATGATGGCGTACTCGCCAAAG	
attB1 CasFB8 FP	TCAAGTTTGTACAAAAAAGCAGGCTATGAACATCCCCGCTCTGGTGGAGAAC	
attB2 CasFB8 RP	CCCACTTTGTACAAGAAAGCTGGGTTTACTTCATGATGGCGTACTCGCCAAAG	
attB1 CasFB9 FP	TCAAGTTTGTACAAAAAAGCAGGCTATGAACATCCCCGCTCTGGTGGAGAAC	
attB2 CasFB9 RP	CCCACTTTGTACAAGAAAGCTGGGTTTACTTCATGATGGCGTACTCGCCAAAG	
attB1 CasFB10 FP	TCAAGTTTGTACAAAAAAGCAGGCTATGAACATCCCCGCTCTGGTGGAGAAC	
attB2 CasFB10 RP	CCCACTTTGTACAAGAAAGCTGGGTTTACTTCATGATGGCGTACTCGCCAAAG	
pC13ccr01	
attB1 Ppe FP	TCAAGTTTGTACAAAAAAGCAGGCTATGGGAAAACCAAATAGAAGTTC	
attB2 Ppe RP	CCCACTTTGTACAAGAAAGCTGGGTTTAAAGGGTTATTTTTAAGTTAAAGC	
attB1 CasFC1 FP	TCAAGTTTGTACAAAAAAGCAGGCTATGGGCAAGCCCAACCGCAGCAGCATCATC	
attB2 CasFC1 RP	CCCACTTTGTACAAGAAAGCTGGGTTTACAGGGTGATCTTCAGGTTGAAGCAG	
attB1 CasFC2 FP	TCAAGTTTGTACAAAAAAGCAGGCTATGGGCAAACCCAACCGCAGCAGCATC	
attB2 CasFC2 RP	CCCACTTTGTACAAGAAAGCTGGGTTTACAGGGTGATTTTCAGGTTGAAGCAG	
attB1 CasFC3 FP	TCAAGTTTGTACAAAAAAGCAGGCTATGGGCAAACCCAACCGCAGCAG	
attB2 CasFC3 RP	CCCACTTTGTACAAGAAAGCTGGGTTTACAGGGTGATTTTCAGGTTGAAGCAGTAG	
attB1 CasFC4 FP	TCAAGTTTGTACAAAAAAGCAGGCTATGGGAAAGCCAAATCGCAGTAGCATC	
attB2 CasFC4 RP	CCCACTTTGTACAAGAAAGCTGGGTTTACAGGGTGATCTTCAGGTTAAAGCAATAATG	
attB1 CasFC5 FP	TCAAGTTTGTACAAAAAAGCAGGCTATGGGAAAGCCAAATCGCAGTAGCATC	
attB2 CasFC5 RP	CCCACTTTGTACAAGAAAGCTGGGTTTACAGGGTGATCTTCAGGTTAAAG	
attB1 CasFC6 FP	TCAAGTTTGTACAAAAAAGCAGGCTATGGGCAAGCCAAATCGCAGTAGCATCATC	
attB2 CasFC6 RP	CCCACTTTGTACAAGAAAGCTGGGTTTACAGGGTGATCTTCAGGTTAAAGCAATAATG	
attB1 CasFC7 FP	TCAAGTTTGTACAAAAAAGCAGGCTATGGGCAAGCCAAACCGCAGTAGCATC	
attB2 CasFC7 RP	CCCACTTTGTACAAGAAAGCTGGGTTTACAGGGTGATCTTCAGGTTAAAGCAATAATG	
attB1 CasFC8 FP	TCAAGTTTGTACAAAAAAGCAGGCTATGGGCAAGCCAAACCGCAGTAGCATC	
attB2 CasFC8 RP	CCCACTTTGTACAAGAAAGCTGGGTTTACAGGGTGATCTTCAGGTTGAAGCAATAATG	
attB1 CasFC9 FP	TCAAGTTTGTACAAAAAAGCAGGCTATGGGCAAGCCAAACCGCAGTAG	
attB2 CasFC9 RP	CCCACTTTGTACAAGAAAGCTGGGTTTACAGGGTGATCTTCAGGTTGAAGCAATAATG	
attB1 CasFC10 FP	TCAAGTTTGTACAAAAAAGCAGGCTATGGGCAAGCCCAACCGCAGCAGCATCATC	
attB2 CasFC10 RP	CCCACTTTGTACAAGAAAGCTGGGTTTACAGGGTGATCTTCAGGTTGAAGCAG	
pC13dcr01	
attB1 RX FP	TCAAGTTTGTACAAAAAAGCAGGCTATGATCGAAAAAAAAAAGTCCTTCGCCAAG	
attB2 RX RP	CCCACTTTGTACAAGAAAGCTGGGTTTAGGAATTGCCGGACACCTTCTTTTTC	
attB1 CasFX1 FP	TCAAGTTTGTACAAAAAAGCAGGCTATGATCGAGAAGAAGAAGAGCTTC	
attB2 CasFX1 RP	CCCACTTTGTACAAGAAAGCTGGGTTTAGCTGTTGCCGCTCACCTTCTTC	
attB1 CasFX2 FP	TCAAGTTTGTACAAAAAAGCAGGCTATGATCGAGAAGAAGAAGTCCTTCGCCAAG	
attB2 CasFX2 RP	CCCACTTTGTACAAGAAAGCTGGGTTTAGGAATTGCCGGACACCTTCTTCTTC	
attB1 CasFX3 FP	TCAAGTTTGTACAAAAAAGCAGGCTATGATCGAGAAAAAAAAGTCCTTCGCCAAG	
attB2 CasFX3 RP	CCCACTTTGTACAAGAAAGCTGGGTTTAGGAATTGCCGGACACCTTCTTTTTC	
attB1 CasFX4 FP	TCAAGTTTGTACAAAAAAGCAGGCTATGATCGAAAAGAAGAAGTCCTTCGCCAAG	
attB2 CasFX4 RP	CCCACTTTGTACAAGAAAGCTGGGTTTAGGAATTGCCGGACACCTTCTTC	
attB1 CasFX5 FP	TCAAGTTTGTACAAAAAAGCAGGCTATGATCGAGAAGAAGAAGTCCTTCGCCAAG	
attB2 CasFX5 RP	CCCACTTTGTACAAGAAAGCTGGGTTTAGGAATTGCCGGACACCTTC	
attB1 CasFX6 FP	TCAAGTTTGTACAAAAAAGCAGGCTATGATCGAGAAGAAGAAGTC	
attB2 CasFX6 RP	CCCACTTTGTACAAGAAAGCTGGGTTTAGGAATTGCCGGACACCTTC	
attB1 CasFX7 FP	TCAAGTTTGTACAAAAAAGCAGGCTATGATCGAGAAGAAGAAGTC	
attB2 CasFX7 RP	CCCACTTTGTACAAGAAAGCTGGGTTTAGGAATTGCCGGACACCTTC	
attB1 CasFX8 FP	TCAAGTTTGTACAAAAAAGCAGGCTATGATCGAGAAGAAGAAGTC	
attB2 CasFX8 RP	CCCACTTTGTACAAGAAAGCTGGGTTTAGGAATTGCCGGACACCTTC	
attB1 CasFX9 FP	TCAAGTTTGTACAAAAAAGCAGGCTATGATCGAGAAGAAGAAGTCCTTC	
attB2 CasFX9 RP	CCCACTTTGTACAAGAAAGCTGGGTTTAGGAATTGCCGGACACCTTC	
attB1 CasFX10 FP	TCAAGTTTGTACAAAAAAGCAGGCTATGATCGAGAAGAAGAAGTC	
attB2 CasFX10 RP	CCCACTTTGTACAAGAAAGCTGGGTTTAGGAATTGCCGGACACCTTC	
pC13cr01 eCFP crRNAs	
C13A eCFP 01 FP	ACTAaggatgggcaccaccccggtgaacagct	
C13A eCFP 01 RP	TTTTagctgttcaccggggtggtgcccatcct	
C13A eCFP 02 FP	ACTAgttcaccagggtgtcgccctcgaacttc	
C13A eCFP 02 RP	TTTTgaagttcgagggcgacaccctggtgaac	
C13B eCFP 01 FP	ACTAcaggatgggcaccaccccggtgaacagctc	
C13B eCFP 01 RP	TTTTgagctgttcaccggggtggtgcccatcctg	
C13B eCFP 02 FP	ACTAcaggatgggcaccaccccggtgaacagctc	
C13B eCFP 02 RP	TTTTgaagttcgagggcgacaccctggtgaaccg	
C13C eCFP 01 FP	ACTAcaggatgggcaccaccccggtgaacagctc	
C13C eCFP 01 RP	TTTTgagctgttcaccggggtggtgcccatcctg	
C13C eCFP 02 FP	ACTAcaggatgggcaccaccccggtgaacagctc	
C13C eCFP 02 RP	TTTTgaagttcgagggcgacaccctggtgaaccg	
C13D eCFP 01 FP	ACTAcaggatgggcaccaccccggtgaacagctc	
C13D eCFP 01 RP	TTTTgagctgttcaccggggtggtgcccatcctg	
C13D eCFP 02 FP	ACTAcaggatgggcaccaccccggtgaacagctc	
C13D eCFP 02 RP	TTTTgaagttcgagggcgacaccctggtgaaccg	
pC13cr eCFP2 mismatch crRNA	
pC13A eCFP2 3 FP	ACTAgtGcaccagggtgtcgccctcgaacttc	
pC13A eCFP2 3 RP	TTTTgaagttcgagggcgacaccctggtgCac	
pC13A eCFP2 6 FP	ACTAgttcaTcagggtgtcgccctcgaacttc	
pC13A eCFP2 6 RP	TTTTgaagttcgagggcgacaccctgAtgaac	
pC13A eCFP2 9 FP	ACTAgttcaccaTggtgtcgccctcgaacttc	
pC13A eCFP2 9 RP	TTTTgaagttcgagggcgacaccAtggtgaac	
pC13A eCFP2 12 FP	ACTAgttcaccagggCgtcgccctcgaacttc	
pC13A eCFP2 12 RP	TTTTgaagttcgagggcgacGccctggtgaac	
pC13A eCFP2 15 FP	ACTAgttcaccagggtgtAgccctcgaacttc	
pC13A eCFP2 15 RP	TTTTgaagttcgagggcTacaccctggtgaac	
pC13A eCFP2 18 FP	ACTAgttcaccagggtgtcgcActcgaacttc	
pC13A eCFP2 18 RP	TTTTgaagttcgagTgcgacaccctggtgaac	
pC13A eCFP2 21 FP	ACTAgttcaccagggtgtcgccctAgaacttc	
pC13A eCFP2 21 RP	TTTTgttcaccagggtgtcgcActcgaacttc	
pC13A eCFP2 24 FP	ACTAgttcaccagggtgtcgccctcgaGcttc	
pC13A eCFP2 24 RP	TTTTgaagCtcgagggcgacaccctggtgaac	
pC13A eCFP2 27 FP	ACTAgttcaccagggtgtcgccctcgaactGc	
pC13A eCFP2 27 RP	TTTTgCagttcgagggcgacaccctggtgaac	
pC13A eCFP2 28 FP	ACTAgttcaccagggtgtcgccctcgaacttA	
pC13A eCFP2 28 RP	TTTTTaagttcgagggcgacaccctggtgaac	
pC13B eCFP2 3 FP	ACTAcggttcaccagggtgtcgccctcgaacGtc	
pC13B eCFP2 3 RP	TTTTgaCgttcgagggcgacaccctggtgaaccg	
pC13B eCFP2 6 FP	ACTAcggttcaccagggtgtcgccctcgGacttc	
pC13B eCFP2 6 RP	TTTTgaagtCcgagggcgacaccctggtgaaccg	
pC13B eCFP2 9 FP	ACTAcggttcaccagggtgtcgcccGcgaacttc	
pC13B eCFP2 9 RP	TTTTgaagttcgCgggcgacaccctggtgaaccg	
pC13B eCFP2 12 FP	ACTAcggttcaccagggtgtcgTcctcgaacttc	
pC13B eCFP2 12 RP	TTTTgaagttcgaggAcgacaccctggtgaaccg	
pC13B eCFP2 15 FP	ACTAcggttcaccagggtgGcgccctcgaacttc	
pC13B eCFP2 15 RP	TTTTgaagttcgagggcgCcaccctggtgaaccg	
pC13B eCFP2 18 FP	ACTAcggttcaccaggAtgtcgccctcgaacttc	
pC13B eCFP2 18 RP	TTTTgaagttcgagggcgacaTcctggtgaaccg	
pC13B eCFP2 21 FP	ACTAcggttcaccCgggtgtcgccctcgaacttc	
pC13B eCFP2 21 RP	TTTTgaagttcgagggcgacacccGggtgaaccg	
pC13B eCFP2 24 FP	ACTAcggttcGccagggtgtcgccctcgaacttc	
pC13B eCFP2 24 RP	TTTTgaagttcgagggcgacaccctggCgaaccg	
pC13B eCFP2 27 FP	ACTAcggCtcaccagggtgtcgccctcgaacttc	
pC13B eCFP2 27 RP	TTTTgaagttcgagggcgacaccctggtgaGccg	
pC13B eCFP2 30 FP	ACTATggttcaccagggtgtcgccctcgaacttc	
pC13B eCFP2 30 RP	TTTTgaagttcgagggcgacaccctggtgaaccA	
pC13C eCFP2 3 FP	ACTAcaTgatgggcaccaccccggtgaacagctc	
pC13C eCFP2 3 RP	TTTTgagctgttcaccggggtggtgcccatcAtg	
pC13C eCFP2 6 FP	ACTAcaggaGgggcaccaccccggtgaacagctc	
pC13C eCFP2 6 RP	TTTTgagctgttcaccggggtggtgcccCtcctg	
pC13C eCFP2 9 FP	ACTAcaggatggAcaccaccccggtgaacagctc	
pC13C eCFP2 9 RP	TTTTgagctgttcaccggggtggtgTccatcctg	
pC13C eCFP2 12 FP	ACTAcaggatgggcaTcaccccggtgaacagctc	
pC13C eCFP2 12 RP	TTTTgagctgttcaccggggtgAtgcccatcctg	
pC13C eCFP2 15 FP	ACTAcaggatgggcaccaTcccggtgaacagctc	
pC13C eCFP2 15 RP	TTTTgagctgttcaccgggAtggtgcccatcctg	
pC13C eCFP2 18 FP	ACTAcaggatgggcaccacccAggtgaacagctc	
pC13C eCFP2 18 RP	TTTTgagctgttcaccTgggtggtgcccatcctg	
pC13C eCFP2 21 FP	ACTAcaggatgggcaccaccccggCgaacagctc	
pC13C eCFP2 21 RP	TTTTgagctgttcGccggggtggtgcccatcctg	
pC13C eCFP2 24 FP	ACTAcaggatgggcaccaccccggtgaGcagctc	
pC13C eCFP2 24 RP	TTTTgagctgCtcaccggggtggtgcccatcctg	
pC13C eCFP2 27 FP	ACTAcaggatgggcaccaccccggtgaacaTctc	
pC13C eCFP2 27 RP	TTTTgagAtgttcaccggggtggtgcccatcctg	
pC13C eCFP2 30 FP	ACTAcaggatgggcaccaccccggtgaacagctA	
pC13C eCFP2 30 RP	TTTTTagctgttcaccggggtggtgcccatcctg	
pC13D eCFP2 1 FP	ACTATaggatgggcaccaccccggtgaacagctc	
pC13D eCFP2 1 RP	TTTTgagctgttcaccggggtggtgcccatcctA	
pC13D eCFP2 3 FP	ACTAcaTgatgggcaccaccccggtgaacagctc	
pC13D eCFP2 3 RP	TTTTgagctgttcaccggggtggtgcccatcAtg	
pC13D eCFP2 6 FP	ACTAcaggaCgggcaccaccccggtgaacagctc	
pC13D eCFP2 6 RP	TTTTgagctgttcaccggggtggtgcccGtcctg	
pC13D eCFP2 9 FP	ACTAcaggatggAcaccaccccggtgaacagctc	
pC13D eCFP2 9 RP	TTTTgagctgttcaccggggtggtgTccatcctg	
pC13D eCFP2 12 FP	ACTAcaggatgggcaTcaccccggtgaacagctc	
pC13D eCFP2 12 RP	TTTTgagctgttcaccggggtgAtgcccatcctg	
pC13D eCFP2 15 FP	ACTAcaggatgggcaccaTcccggtgaacagctc	
pC13D eCFP2 15 RP	TTTTgagctgttcaccgggAtggtgcccatcctg	
pC13D eCFP2 18 FP	ACTAcaggatgggcaccacccAggtgaacagctc	
pC13D eCFP2 18 RP	TTTTgagctgttcaccTgggtggtgcccatcctg	
pC13D eCFP2 21 FP	ACTAcaggatgggcaccaccccggCgaacagctc	
pC13D eCFP2 21 RP	TTTTgagctgttcGccggggtggtgcccatcctg	
pC13D eCFP2 24 FP	ACTAcaggatgggcaccaccccggtgaGcagctc	
pC13D eCFP2 24 RP	TTTTgagctgCtcaccggggtggtgcccatcctg	
pC13D eCFP2 27 FP	ACTAcaggatgggcaccaccccggtgaacaTctc	
pC13D eCFP2 27 RP	TTTTgagAtgttcaccggggtggtgcccatcctg	
pC13D eCFP2 30 FP	ACTAcaggatgggcaccaccccggtgaacagctA	
pC13D eCFP2 30 RP	TTTTTagctgttcaccggggtggtgcccatcctg	
pC13A 3+6 FP	ACTAgtGcaTcagggtgtcgccctcgaacttc	
pC13A 3+6 RP	TTTTgaagttcgagggcgacaccctgAtgCac	
pC13A 3+15 FP	ACTAgtGcaccagggtgtAgccctcgaacttc	
pC13A 3+15 RP	TTTTgaagttcgagggcTacaccctggtgCac	
pC13A 3+28 FP	ACTAgtGcaccagggtgtcgccctcgaacttA	
pC13A 3+28 RP	TTTTTaagttcgagggcgacaccctggtgCac	
pC13A 3+6+15 FP	ACTAgtGcaTcagggtgtAgccctcgaacttc	
pC13A 3+6+15 RP	TTTTgaagttcgagggcTacaccctgAtgCac	
pC13A 3+6+28 FP	ACTAgtGcaTcagggtgtcgccctcgaacttA	
pC13A 3+6+28 RP	TTTTTaagttcgagggcgacaccctgAtgCac	
pC13A 3+6+15+28 FP	ACTAgtGcaTcagggtgtAgccctcgaacttA	
pC13A 3+6+15+28 RP	TTTTTaagttcgagggcTacaccctgAtgCac	
pC13B 3+6 FP	ACTAcggttcaccagggtgtcgccctcgGacGtc	
pC13B 3+6 RP	TTTTgaCgtCcgagggcgacaccctggtgaaccg	
pC13B 3+15 FP	ACTAcggttcaccagggtgGcgccctcgaacGtc	
pC13B 3+15 RP	TTTTgaCgttcgagggcgCcaccctggtgaaccg	
pC13B 3+30 FP	ACTATggttcaccagggtgtcgccctcgaacGtc	
pC13B 3+30 RP	TTTTgaCgttcgagggcgacaccctggtgaaccA	
pC13B 3+6+15 FP	ACTAcggttcaccagggtgGcgccctcgGacGtc	
pC13B 3+6+15 RP	TTTTgaCgtCcgagggcgCcaccctggtgaaccg	
pC13B 3+6+30 FP	ACTATggttcaccagggtgtcgccctcgGacGtc	
pC13B 3+6+30 RP	TTTTgaCgtCcgagggcgacaccctggtgaaccA	
pC13B 3+6+15+30 FP	ACTATggttcaccagggtgGcgccctcgGacGtc	
pC13B 3+6+15+30 RP	TTTTgaCgtCcgagggcgCcaccctggtgaaccA	
pC13C 3+6 FP	ACTAcaTgaGgggcaccaccccggtgaacagctc	
pC13C 3+6 RP	TTTTgagctgttcaccggggtggtgcccCtcAtg	
pC13C 3+15 FP	ACTAcaTgatgggcaccaTcccggtgaacagctc	
pC13C 3+15 RP	TTTTgagctgttcaccgggAtggtgcccatcAtg	
pC13C 3+30 FP	ACTAcaTgatgggcaccaccccggtgaacagctA	
pC13C 3+30 RP	TTTTTagctgttcaccggggtggtgcccatcAtg	
pC13C 3+6+15 FP	ACTAcaTgaGgggcaccaTcccggtgaacagctc	
pC13C 3+6+15 RP	TTTTgagctgttcaccgggAtggtgcccCtcAtg	
pC13C 3+6+30 FP	ACTAcaTgaGgggcaccaccccggtgaacagctA	
pC13C 3+6+30 RP	TTTTTagctgttcaccggggtggtgcccCtcAtg	
pC13C 3+6+15+30 FP	ACTAcaTgaGgggcaccaTcccggtgaacagctA	
pC13C 3+6+15+30 RP	TTTTTagctgttcaccgggAtggtgcccCtcAtg	
pC13D 3+6 FP	ACTAcaTgaCgggcaccaccccggtgaacagctc	
pC13D 3+6 RP	TTTTgagctgttcaccggggtggtgcccGtcAtg	
pC13D 3+15 FP	ACTAcaTgatgggcaccaTcccggtgaacagctc	
pC13D 3+15 RP	TTTTgagctgttcaccgggAtggtgcccatcAtg	
pC13D 3+30 FP	ACTAcaTgatgggcaccaccccggtgaacagctA	
pC13D 3+30 RP	TTTTTagctgttcaccggggtggtgcccatcAtg	
pC13D 3+6+15 FP	ACTAcaTgaCgggcaccaTcccggtgaacagctc	
pC13D 3+6+15 RP	TTTTgagctgttcaccgggAtggtgcccGtcAtg	
pC13D 3+6+30 FP	ACTAcaTgaCgggcaccaccccggtgaacagctA	
pC13D 3+6+30 RP	TTTTTagctgttcaccggggtggtgcccGtcAtg	
pC13D 3+6+15+30 FP	ACTAcaTgaCgggcaccaTcccggtgaacagctA	
pC13D 3+6+15+30 RP	TTTTTagctgttcaccgggAtggtgcccGtcAtg	
dCasFX Fer1HCH RA crRNA	
dCasFX RA cr01 FP	ACTATGTGATACACACGTCCTCAATATGGGTATA	
dCasFX RA cr01 RP	TTTTTATACCCATATTGAGGACGTGTGTATCACA	
dCasFX RA cr02 FP	ACTAGCTCCGAACGGCGCACAAAACACTTTTAAG	
dCasFX RA cr02 RP	TTTTCTTAAAAGTGTTTTGTGCGCCGTTCGGAGC	
dCasFX RA cr03 FP	ACTAAGAAGGCGTCGCCTGCTTCAATTTGATGGG	
dCasFX RA cr03 RP	TTTTCCCATCAAATTGAAGCAGGCGACGCCTTCT	
dCasFX RA cr04 FP	ACTACATCTTTGATCGTCGAACGTAGTCTTTACA	
dCasFX RA cr04 RP	TTTTTGTAAAGACTACGTTCGACGATCAAAGATG	
dCasFX RA cr05 FP	ACTACTGGTAGGAGGCGTTGATCTCCTCCTGGAT	
dCasFX RA cr05 RP	TTTTATCCAGGAGGAGATCAACGCCTCCTACCAG	
dCasFX RA cr06 FP	ACTATCGGCACATTGATCAGATCGCTGACTCCCT	
dCasFX RA cr06 RP	TTTTAGGGAGTCAGCGATCTGATCAATGTGCCGA	
dCasFX RA cr07 FP	ACTAGTGGAGCTGCTCCTCCAGATAGACACCGGT	
dCasFX RA cr07 RP	TTTTACCGGTGTCTATCTGGAGGAGCAGCTCCAC	
dCasFX RA cr08 FP	ACTAGGGCTGACAGATAACAGATAACTCGACTGG	
dCasFX RA cr08 RP	TTTTCCAGTCGAGTTATCTGTTATCTGTCAGCCC	
dCasFX RA cr09 FP	ACTAGCATAATGTGGAGCCCCCTCCCGAGGTGTA	
dCasFX RA cr09 RP	TTTTTACACCTCGGGAGGGGGCTCCACATTATGC	
mitochondrial encoded genes	
COXI cr01 FP	ACTAAAGATGTTCCAACTATTCCAGCTCAAGCTC	
COXI cr01 RP	TTTTGAGCTTGAGCTGGAATAGTTGGAACATCTT	
COXI cr02 FP	ACTAGAGGTGGATAAACAGTTCATCCTGTCCCAG	
COXI cr02 RP	TTTTCTGGGACAGGATGAACTGTTTATCCACCTC	
COXI cr03 FP	ACTAGAGGATTAACAGGAGTTGTTTTAGCTAATT	
COXI cr03 RP	TTTTAATTAGCTAAAACAACTCCTGTTAATCCTC	
COXI cr04 FP	ACTAATAATGAAATAGTTGATCCAATAGTTGATA	
COXI cr04 RP	TTTTTATCAACTATTGGATCAACTATTTCATTAT	
COXII cr01 FP	ACTAGAAGCTCTATCTTGTAAACCTAAATTAGCT	
COXII cr01 RP	TTTTAGCTAATTTAGGTTTACAAGATAGAGCTTC	
COXII cr02 FP	ACTAATAATATAAATATTAAATATCCCACCAATA	
COXII cr02 RP	TTTTTATTGGTGGGATATTTAATATTTATATTAT	
COXII cr03 FP	ACTACATTTGTTGGAATTATATATGAATCAAATT	
COXII cr03 RP	TTTTAATTTGATTCATATATAATTCCAACAAATG	
COXII cr04 FP	ACTAAATTAGTTTGATTTAATCGTCCAGGTGTAC	
COXII cr04 RP	TTTTGTACACCTGGACGATTAAATCAAACTAATT	
COXI RNAi 2 FP	CTAGCAGTCGAGCTGAATTAGGACATCCTAGTTATATTCAAGCATAGGATGTCCTAATTCAGCTCGGCG	
COXI RNAi 2 RP	AATTCGCCGAGCTGAATTAGGACATCCTATGCTTGAATATAACTAGGATGTCCTAATTCAGCTCGACTG	
COXI RNAi 3 FP	CTAGCAGTAGGTGCTCCTGATATAGCATTAGTTATATTCAAGCATAATGCTATATCAGGAGCACCTGCG	
COXI RNAi 3 RP	AATTCGCAGGTGCTCCTGATATAGCATTATGCTTGAATATAACTAATGCTATATCAGGAGCACCTACTG	
COXI RNAi 4 FP	CTAGCAGTAGCTGGGACAGGATGAACTGTAGTTATATTCAAGCATACAGTTCATCCTGTCCCAGCTGCG	
COXI RNAi 4 RP	AATTCGCAGCTGGGACAGGATGAACTGTATGCTTGAATATAACTACAGTTCATCCTGTCCCAGCTACTG	
COXII RNAi 2 FP	CTAGCAGTCATGATCATGCATTATTAATTAGTTATATTCAAGCATAATTAATAATGCATGATCATGGCG	
COXII RNAi 2 RP	AATTCGCCATGATCATGCATTATTAATTATGCTTGAATATAACTAATTAATAATGCATGATCATGACTG	
COXII RNAi 3 FP	CTAGCAGTAGCTGCTGATGTTATTCATTTAGTTATATTCAAGCATAAATGAATAACATCAGCAGCTGCG	
COXII RNAi 3 RP	AATTCGCAGCTGCTGATGTTATTCATTTATGCTTGAATATAACTAAATGAATAACATCAGCAGCTACTG	
COXII RNAi 4 FP	CTAGCAGTTCTGTGGAGCTAATCATAGATAGTTATATTCAAGCATATCTATGATTAGCTCCACAGAGCG	
COXII RNAi 4 RP	AATTCGCTCTGTGGAGCTAATCATAGATATGCTTGAATATAACTATCTATGATTAGCTCCACAGAACTG	
FREPAIRv2 crRNAs	
eCFP* 50cr02 FP	ACTAggCTAccagggcacgggcagcttgccggtggtgcagatgaacttcagggt	
eCFP* 50cr02 RP	TTTTaccctgaagttcatctgcaccaccggcaagctgcccgtgccctggTAGcc	
eCFP* 50cr10 FP	ACTAgagggtgggCTAccagggcacgggcagcttgccggtggtgcagatgaact	
eCFP* 50cr10 RP	TTTTagttcatctgcaccaccggcaagctgcccgtgccctggTAGcccaccctc	
eCFP* 50cr18 FP	ACTAgtggtcacgagggtgggCTAccagggcacgggcagcttgccggtggtgca	
eCFP* 50cr18 RP	TTTTtgcaccaccggcaagctgcccgtgccctggTAGcccaccctcgtgaccac	
eCFP* 50cr26 FP	ACTAaggtcagggtggtcacgagggtgggCTAccagggcacgggcagcttgccg	
eCFP* 50cr26 RP	TTTTcggcaagctgcccgtgccctggTAGcccaccctcgtgaccaccctgacct	
eCFP* 50cr34 FP	ACTAcacgccccaggtcagggtggtcacgagggtgggCTAgggcacgggcagct	
eCFP* 50cr34 RP	TTTTagctgcccgtgcccTAGcccaccctcgtgaccaccctgacctggggcgtg	
eCFP* 50cr42 FP	ACTAaagcactgcacgccccaggtcagggtggtcacgagggtgggCTAgggcac	
eCFP* 50cr42 RP	TTTTgtgcccTAGcccaccctcgtgaccaccctgacctggggcgtgcagtgctt	
eCFP* 50cr50 FP	ACTAagcggctgaagcactgcacgccccaggtcagggtggtcacgagggtgggC	
eCFP* 50cr50 RP	TTTTGcccaccctcgtgaccaccctgacctggggcgtgcagtgcttcagccgct	
eCFP* 30cr26 FP	ACTAaggtcagggtggtcacgagggtgggCTAcc	
eCFP* 30cr26 RP	TTTTggTAGcccaccctcgtgaccaccctgacct	
eCFP* 40cr26 FP	ACTAaggtcagggtggtcacgagggtgggCTAccagggcacggg	
eCFP* 40cr26 RP	TTTTcccgtgccctggTAGcccaccctcgtgaccaccctgacct	
eCFP* 60cr26 FP	ACTAaggtcagggtggtcacgagggtgggCTAccagggcacgggcagcttgccggtggtgcaga	
eCFP* 60cr26 RP	TTTTtctgcaccaccggcaagctgcccgtgccctggTAGcccaccctcgtgaccaccctgacct	
eCFP* 70cr26 FP	ACTAaggtcagggtggtcacgagggtgggCTAccagggcacgggcagcttgccggtggtgcagatgaacttcag	
eCFP* 70cr26 RP	TTTTctgaagttcatctgcaccaccggcaagctgcccgtgccctggTAGcccaccctcgtgaccaccctgacct	
eCFP* 80cr26 FP	ACTAaggtcagggtggtcacgagggtgggCTAccagggcacgggcagcttgccggtggtgcagatgaacttcagcaagctgacc	
eCFP* 80cr26 RP	TTTTggtcagcttgctgaagttcatctgcaccaccggcaagctgcccgtgccctggTAGcccaccctcgtgaccaccctgacct	
mutagenesis primers	
dCasFX RH5 mut FP	TCCAGGATCTCCAGGACCTGGCTCTACAACCTCGATAAGAACCTC	
dCasFX RH5 Mut RP	CTCTTCTTCGTTGTTTGCGACCACCCAGTGTGCCAGTCCGCTCAG	
dCasFX RH3 Mut FP	CCACGCCTATATCAACGACATTGCCGAGGTCAATTCCTACTTCCAACTGTAC	
dCasFX RH3 Mut RP	ACATACCTGGCCACTTCCAGTGCGACGGCCTTGTTTGCGAACAGGGTACATG	
mtCasFX Mut FP	TCAAGTTTGTACAAAAAAGCAGGCTATGAGTGACAATTTTTCAAGAACACCATATATCGAAAAGAAGAAGTCCTTC	
mtCasFX Mut RP	CCCACTTTGTACAAGAAAGCTGGGTTTAGGAATTGCCGGACACCTTCTTC	
FREPAIRv2 F1 FP	CTCCGCGGCCGCCCCCTTCACCATGATCGAAAAAAAAAAGTCCTTC	
FREPAIRv2 F1 RP	cagtctttcaagtggaggcagctgcaggcttccGGAATTGCCGGACACCTTC	
FREPAIRv2 F2 FP	tgcctccacttgaaagactgacactgggatccggaggaggtggaagccagctgcatttac	
FREPAIRv2 F2 RP	TGGGTCGGCGCGCCCACCCTTttacgtgagtgagaactggtcctgctc	
eCFP W57* Mut FP	agctgcccgtgcccTAAcccaccctcgtgaccac	
eCFP W57* Mut RP	tgccggtggtgcagatgaacttcagggtcag	
crRNA transgenes	
pC13B F1 RP	CTTCGTCCCAGGAAGACATggtgGCATCGGCCGGGAATCGAAC	
pC13B F1 FP	gtcagcggtttcgtgacgaagctccaag	
pC13B F2 RP	cttggagcttcgtcacgaaaccgctgac	
pC13B F2 FP	CACTTTGAAGGGTATTCACAACttttttgcctacctggagcctgagag	
pC13B Middle FP	ccATGTCTTCCTGGGACGAAGACAAGTTGGGACTGCTCTCACTTTGAAGGGTATTCACAACtaacaaagcaccagtggtctagtggtagaatagtaccctgccacggtacagacccGGGTTCGATTCCCGGCTGGTGCAcaccATGTCTTCCTGGGACGAAGACAAGTTGGGACTGCTCTCACTTTGAAGGGTATTCACA	
pC13B Middle RP	TGTGAATACCCTTCAAAGTGAGAGCAGTCCCAACTTGTCTTCGTCCCAGGAAGACATggtgTGCACCAGCCGGGAATCGAACCCgggtctgtaccgtggcagggtactattctaccactagaccactggtgctttgttaGTTGTGAATACCCTTCAAAGTGAGAGCAGTCCCAACTTGTCTTCGTCCCAGGAAGACATgg	
pC13X F1 RP	CCGACCAGTTGGTAGGGGTTGCATCGGCCGGGAATCGAAC	
pC13X F1 FP	gtcagcggtttcgtgacgaagctccaag	
pC13X F2 RP	cttggagcttcgtcacgaaaccgctgac	
pC13X F2 FP	ACggGTCTTCgaGAAGACctttttttgcctacctggagcctgagagttg	
pC13X Middle FP	AACCCCTACCAACTGGTCGGGGTTTGAAACggGTCTTCgaGAAGACcttaacaaagcaccagtggtctagtggtagaatagtaccctgccacggtacagacccGGGTTCGATTCCCGGCTGGTGCAACCCCTACCAACTGGTCGGGGTTTGAAACggGTCTTCgaGAAGACct	
pC13X Middle RP	agGTCTTCtcGAAGACccGTTTCAAACCCCGACCAGTTGGTAGGGGTTGCACCAGCCGGGAATCGAACCCgggtctgtaccgtggcagggtactattctaccactagaccactggtgctttgttaagGTCTTCtcGAAGACccGTTTCAAACCCCGACCAGTTGGTAGGGGTT	
dib crRNA  pC13X FP	AAACTGGGCAGTAAAAGACTGCAGACGAGCTCCA	
dib crRNA pC13X RP	AAAATGGAGCTCGTCTGCAGTCTTTTACTGCCCA	
dib crRNA pC13B FP	CACCTGGGCAGTAAAAGACTGCAGACGAGCTCCA	
dib crRNA pC13B RP	CAACTGGAGCTCGTCTGCAGTCTTTTACTGCCCA	
phm crRNA pC13X FP	AAACTGGAGCGCCGGCAGCGGATTCACTTCCGAC	
phm crRNA pC13X RP	AAAAGTCGGAAGTGAATCCGCTGCCGGCGCTCCA	
phm crRNA pC13B FP	CACCTGGAGCGCCGGCAGCGGATTCACTTCCGAC	
phm crRNA pC13B RP	CAACGTCGGAAGTGAATCCGCTGCCGGCGCTCCA	
IRP1A crRNA C13X FP	AAACTGGAGCGCCGGCAGCGGATTCACTTCCGAC	
IRP1A crRNA C13X RP	AAAAGTCGGAAGTGAATCCGCTGCCGGCGCTCCA	
IRP1A crRNA C13B FP	CACCTGGAGCGCCGGCAGCGGATTCACTTCCGAC	
IRP1A crRNA C13B RP	CAACGTCGGAAGTGAATCCGCTGCCGGCGCTCCA	
dib cr IRP1A cr C13X FP	AACCCCTACCAACTGGTCGGGGTTTGAAACTGGGCAGTAAAAGACTGCAGACGAGCTCCAtaacaaagcaccagtggtctag	
dib cr IRP1A cr C13X RP	aggctccaggtaggcaaaaaaGTCGGAAGTGAATCCGCTGCCGGCGCTCCAGTTTCAAACCCCGACCAGTTG	
qPCR primers	
eCFP qPCR FP	gaagcgcgatcacatggt	
eCFP qPCR RP	ccatgccgagagtgatcc	
DsRed qPCR FP	gaagggcgagatccacaag	
DsRed qPCR RP	ggacttgaactccaccaggta	
rp49 qPCR FP	CGGATCGATATGCTAAGCTGT	
rp49 qPCR RP	CGACGCACTCTGTTGTCG	
COXI qPCR FP	tgacttctacctcctgctctttc	
COXI qPCR RP	gcggatagaggtggataaacag	
COXII qPCR FP	ccgagtagttttacccataaactca	
COXII qPCR RP	aagcaggtactgttcaagaatgaat	
dib qPCR FP	gtgaccaaggagttcattagatttc	
dib qPCR RP	ccaaaggtaagcaaacaggttaat	
