# Supplementary material for: A versatile toolkit for CRISPR-Cas13-based RNA manipulation in Drosophila
Source: Genome Biol. 2020 Nov 17;21:279. doi: 10.1186/s13059-020-02193-y (PMC7670108; doi:10.1186/s13059-020-02193-y)
Supplement: Supplementary file 6 — Additional file 6. Supplemental methods S1. Cloning procedures for CRISPR/Cas13-crRNA vectors. [file 13059_2020_2193_MOESM6_ESM.rtf]

crRNA cloning protocol

1. Designing the construct:
1a. Choosing target site(s) for crRNA:
- Evaluate target transcript secondary structure using either or both of the following online tools:
	RNAfold: http://rna.tbi.univie.ac.at/cgi-bin/RNAWebSuite/RNAfold.cgi
	RNAstructure: https://rna.urmc.rochester.edu/RNAstructureWeb/Servers/Predict1/Predict1.html
- Optional: one can also use siRNA design tool RNAxs (http://rna.tbi.univie.ac.at/cgi-bin/RNAxs/RNAxs.cgi) to find the regions of transcripts with good accessibility.

1b. Sequencing to verify if your target site(s) exists:
- Extract DNA from fly stocks used to perform embryo injection, G0 cross, and balancing to ensure target site(s) all exists in those lines. 
	For our case, we used the following: 
	. Lines to be injected: 		# 25709 (Bloomington) (attP site on 2nd chromosome)
 					#25710 (Bloomington) (attP site on 3rd chromosome) 
	. Line for G0 cross: 		TBX-0004 (Japan National Institute of Genetics_NIG)
	. Lines for balancing:		TBX-0007 (NIG)
					TBX-0008 (NIG)
					TBX-0009 (NIG)
					TBX-00103
- Design PCR primers that cover region of interest, ensuring that target sites are inside that region.
- Perform PCR + sequencing to verify the accuracy of your target site(s).
2. Design crRNA primers:
2a. Single crRNA:
- Order standard desalted oligos:
	For pC13B: 
		Forward primer: 	5' - CACC (antisense sequence)30 - 3'
		Reverse primer: 	5' - CAAC (sense sequence)30 - 3'
	For pC13X:
		Forward primer:	5' - AAAC (antisense sequence)30 - 3'
		Reverse primer: 	5' - AAAA (sense sequence)30 - 3'

2b. 2 crRNAs:
- Order oligos:
	For pC13B
		Forward primer:		
			5' - GTTCGATTCCCGGCCGATGCcacc (antisense sequence 1)30 GTTGGGACTGCTCTCACTTTG - 3'
		
		Reverse primer:	
			5' - CAAAGTGAGAGCAGTCCCAAC (sense sequence 2)30 ggtgTGCACCAGCCGGGAATC - 3'

	For pC13X
		Forward primer:
			5' - AACCCCTACCAACTGGTCGGGGTTTGAAAC (antisense sequence 1)30 taacaaagcaccagtggtctag - 3'
		
		Reverse primer:
			5' - aggctccaggtaggcaaaaaa (sense sequence 2)30 GTTTCAAACCCCGACCAGTTG - 3'

3c. ≥ 3 crRNAs
- Order oligos:
	For pC13B
		Distal forward primer:		
			5' - GTTCGATTCCCGGCCGATGCcacc (antisense sequence 1)30 GTTGGGACTGCTCTCACTTTG - 3'
		
		F1 reverse primer:
			5' - (sense sequence 2)30 ggtgTGCACCAGCCGGGAATC - 3'
		
		F2 forward primer:
			5' - (antisense sequence 2)30 GTTGGGACTGCTCTCACTTTGAAGGGTATTCACAACtaacaaagcaccagtggtc - 3'
		
		F2 reverse primer:
			5' - (sense sequence 2)30 ggtgTGCACCAGCCGGGAATC- 3'

		Fn forward primer:
			5' - (antisense sequence (n-1))30 GTTGGGACTGCTCTCACTTTGAAGGGTATTCACAACtaacaaagcaccagtggtc - 3'

		Distal reverse primer: 
			5' - CAAAGTGAGAGCAGTCCCAAC (sense sequence n)30 ggtgTGCACCAGCCGGGAATC - 3'

	For pC13X
		Distal forward primer:		
			5' - AACCCCTACCAACTGGTCGGGGTTTGAAAC (antisense sequence 1)30 taacaaagcaccagtggtctag - 3'
		
		F1 reverse primer:
			5' - (sense sequence 2)30 GTTTCAAACCCCGACCAGTTG- 3'
		
		F2 forward primer:
			5' - (antisense sequence 2)30 taacaaagcaccagtggtctagtg - 3'
		
		F2 reverse primer:
			5' - (sense sequence 2)30 GTTTCAAACCCCGACCAGTTG- 3'

		Fn forward primer:
			5'-(antisense sequence (n-1))30 taacaaagcaccagtggtctagtg-3'

		Distal reverse primer: 
			5' - aggctccaggtaggcaaaaaa (sense sequence n)30 GTTTCAAACCCCGACCAGTTG - 3'

3. Prepare backbone:
- Digest pC13B or pC13X plasmid with BbsI restriction enzyme:
reagents	amount added	
10x CutSmart buffer	2.0 μl	
plasmid	X (5 μg)	
BbsI HF (NEB R3539S)	1.0 μl	
nuclease-free water	Y μl (to total volume of 20 μl)	
total	20.0 μl	

- Incubate reaction overnight at 37°C.
- Purify digested backbone using either gel extraction or magnetic beads method.
- Elute backbone to the final concentration of 50-100 ng/μl in nuclease-free water.

4. Prepare crRNA fragment(s):
4a. Single crRNA cloning
- Resuspend oligos in nuclease-free water to a concentration of 100 μM
- Phosphorylation and annealing reaction
reagents	amount added	
forward primer (100 μM)	1.0 μl	
reverse primer (100 μM)	1.0 μl	
10x T4 ligation buffer (NEB)	1.0 μl	
nuclease-free water 	6.0 μl	
T4 Polynucleotide Kinase (NEB M0201S)	1.0 μl	
total	10.0 μl	

- Incubate reaction in a thermocycler:
temperature (0C)	time	
37	30 minutes	
95	5 minutes	
ramp down to 250C at the rate 50C/minute	
4	forever	

- Ligation reaction is done at room temperature for at least 1 hour:
reagents	amount added	
Bbsi-digested plasmid backbone 	1.0 μl (50-100 ng)	
annealed oligos	1.0 μl	
10X T4 ligation buffer (NEB)	1.0 μl	
nuclease-free water	6.0 μl	
T4 DNA ligase (NEB M0202S)	1.0 μl	
total	10.0 μl	

- Proceed to transformation.

4b. Double or multiple crRNAs
- Set up PCR reaction using high fidelity polymerase
reagents	amount added (μl)	
nuclease-free water	10.75	
5x Q5 reaction buffer	5.0	
5x Q5 high GC enhancer buffer	5.0	
10mM dNTPs	0.5	
10μM forward primer	1.25	
10μM reverse primer	1.25	
crRNA plasmid backbone (10ng/μl)	1.0	
Q5 high fidelity DNA polymerase 	0.25	
total	25.0	

- Run PCR in a thermocycler
step	temperature (0C)	time	
1	98	0:30	
2	98	0:15	
3	annealing temperature	0:15	
4	72	30 sec / kb	
go to step 2 for 25-35 cycles	
5	72	2:00	
6	4	forever	

- Check PCR on electrophoresis gel
- Perform DpnI digestion overnight at 370C or gel extraction to eliminate original template.

5. Generation of final crRNA construct (2 or more crRNAs)
- Set up Gibson assembly reaction:
	. Calculate amount of each fragment: : https://nebiocalculator.neb.com/#!/ligation
	. Recommended ratio for PCR fragment : digested backbone = 3:1 to 5:1 (molar ratio)
	. Prepare Gibson reaction: This is an example of Gibson assembly for 4 PCR fragments into the same backbone
reagents	amount added (μl)	
PCR fragment 1	X	
PCR fragment 2	Y	
PCR fragment 3	Z	
PCR fragment 4	W	
digested backbone	1.0 (50-100ng)	
1.33x Gibson assembly	3* (X + Y + Z + W +1)	
	. Incubate reaction in a thermocycler at 500C for 1-4 hours.
- Proceed to transformation.
- To screen for positive cloning, use the following primer:
		5' - acgttttataacttatgcccctaag - 3'
