# Supplementary material for: A versatile toolkit for CRISPR-Cas13-based RNA manipulation in Drosophila
Source: Genome Biol. 2020 Nov 17;21:279. doi: 10.1186/s13059-020-02193-y (PMC7670108; doi:10.1186/s13059-020-02193-y)
Supplement: Supplementary file 7 — Additional file 7. Review history. [file 13059_2020_2193_MOESM7_ESM.docx]

Review History

**First round of review**

**Reviewer 1**

**Are you able to assess all statistics in the manuscript, including the appropriateness of statistical tests used?** Yes, and I have assessed the statistics in my report.

**Comments to author:**

In this interesting manuscript, Huynh and colleagues report on the development of toolkits for using CRISPR/Cas13 in Drosophila cell culture and in vivo. To adapt and optimize CRISPR/Cas13 for Drosophila, the authors tested 40 Cas13 variants representing the four identified Cas13 subtypes. After identifying the most active variants in cell culture, the authors went on to assess collateral cleavage, which has been observed in some systems, and found none. They next assessed fidelity, and identified two Cas13 variants that exhibited no tolerance for mismatches between the crRNA and target sequences. They then generate and test three Cas13 variants to expand potential applications: 1) a nuclease-dead version that they demonstrate can be used for RNA pull downs, 2) a mitochondria-targeted version capable of efficiently knocking down mitochondrial transcripts, and 3) an ADAR2 fusion for targeted RNA editing. Finally, they build on these findings to move in vivo. The authors generate multiple Cas13 Drosophila lines and demonstrate efficient knock down of target transcripts that recapitulate known mutant phenotypes. Overall this work is very thorough and will be of great interest to the entire Drosophila research community, as well as researchers in other models seeking to adapt Cas13 for in vivo RNA targeting. The authors establish valuable toolsets and define the parameters for optimal Cas13-based targeting. Their comprehensive analysis of Cas13 variants with widely varying activity levels also provides a valuable resource for fine tuning transcript knock down. Concerns are minor and can largely be addressed through changes to the text and figures.

Specific comments:
1. All statistical tests need to be described and justified (e.g., are data normally distributed). Figure 5 lacks statistics.

2. A bioRxiv paper (Buchman et al.) describing the use of the CasRx in flies was posted in April. They observed significant toxic effects from expressing Cas13 alone. Experiments addressing potential toxicity in flies should be included. It would also be ideal for the authors to cite this work and address in their discussion. Note that I don’t think it affects the significance or novelty of this manuscript since Buchman et al. only investigated one Cas13 variant with much less success due to general toxicity and low efficiency.

3. For the Cas13 pull down experiments, the argument against also looking directly at mRNA (lines 293-298 ) is not compelling and the authors should show both target mRNA and protein. The text and figure should also make clear that the HA tag on Cas13 was used for pull down.

4. In the experiments targeting multiple transcripts (Figure 6G), did the authors also conduct qPCR? It would be very interesting to quantitatively compare the efficiency of knockdown to single targeting experiments.

5. Lines 423-425 are confusing. What does ‘sequence deviations’ refer to? Do these changes go beyond nearby A->I modifications? This should be clarified and, if the answer is yes, addressed.

6. Overall the writing was very clear and easy to follow. However, the text could benefit from trimming. The introduction and discussion/conclusion in particular can be focused to hone in on key points and remove redundancies. The results section on the REPAIR construct can be shortened a lot.

7. The authors do not demonstrate that the addition of NLS or NES sequences successfully modulate the subcellular localization of the Cas13 variants, limiting their ability to conclude that activity is independent of subcellular localization. This caveat should be addressed or complementary imaging data should be provided (Lines 193-196 and Figure S3).

8. While dCasFX could in principle be used to determine subcellular localization of target RNAs, the authors haven’t shown this and should temper statements accordingly (Lines 320-321 and 519).

9. The Cas13 variant naming is a bit confusing. It might be helpful in the paragraph starting on line 112 to explain why d-subtype variants are named with an X.

10. There are some missing references:
Lines 89-92: The original 2013 Drosophila CRISPR-Cas9 work from several labs is omitted.
Lines 115-117: The references for the relevant mammalian cell culture should be included here.

11. Occasionally, unsupported or overly strong statements are made about various related technologies, e.g. meganucleases, zinc finger nucleases and transcription activator-like effector nuclease on lines 62-65; RNAi and CRISPRi on lines  lines 80-81; and CRISPR-based techniques on line 92.

12. The graphical representation of lethality rates from Table S3 might be more informative in place of or in addition to the phenotypic imaging provided in Figure 6. The images in these panels may be better suited as supplementary data.  

13. On Line 541, the modifier ‘real’ should be removed.

14. Line 43 should read CRISPR-associated proteins instead of CRISPR-associated proteins.

**Reviewer 2**

**Are you able to assess all statistics in the manuscript, including the appropriateness of statistical tests used?** Yes, and I have assessed the statistics in my report.

**Comments to author:**

The work by Huynh et al.  entitled " A versatile tool-kit for CRISPR-Cas13-based RNA manipulations in Drosophila" demonstrates the interrogation of Cas13 variants to identify a potent variant, Cas13d, for targeted knock down of RNA transcripts, in vivo and in cell culture.
In this study the authors tested 40 Cas13 variants of the four subtypes of the type VI CRISPR system. Cas13d exhibited around 82% efficiency in knocking down the target transcripts whereas CasFX4 variant showed 90% knock-down efficiency. Interestingly, their data have confirmed a recent report that the nuclear of cytoplasmic localization of the Cas13 variant does not seem to influence the efficiency of targeting the RNA transcripts. And consistent with studies in mammalian and plant systems, Cas13 variants see to lack collateral activities in vivo.
Furthermore, CasFX4 and CasFX8 exhibit low off-target activities. And these variants were tested for RNA knock-down, RNA- recognition and RNA editing as well as targeting mitochondrial RNA transcripts. The authors have nicely shown different applications of the CRISPR- Cas13 system beyond RNA interference which will be key for the future applications of D. M research. This toolkit will definitely be useful to the community.
Similar toolkits are highly needed for other eukaryotic species to expedite basic and translational research activities.

The manuscript is very well written, and the topic is timely. This manuscript builds on prior work from this lab on the use of CRISPR systems for the manipulations of the Drosophila genome. And all methods are relevant to this research project and to the aims of this study. Most importantly, the conclusions are supported by the experiments and the data. If the authors produce a tool-kit, this implies that the reagents will be able from Addgene or similar platform for distribution to the Drosophila labs. I think the authors need to include a statemen on how they are planning to share these reagents. Although the work demonstrates the feasibility of using CARISPR-Cas13 systems specifically CasRx for different RNA manipulations, it is very useful for the community and such toolkits for other systems will be equally eligible to receive similar attention. This manuscript is of great interest to D.M research labs and will impact the basic and applied research in this model system.

**Authors Response**

Reviewer #1: In this interesting manuscript, Huynh and colleagues report on the development of toolkits for using CRISPR/Cas13 in Drosophila cell culture and in vivo. To adapt and optimize CRISPR/Cas13 for Drosophila, the authors tested 40 Cas13 variants representing the four identified Cas13 subtypes. After identifying the most active variants in cell culture, the authors went on to assess collateral cleavage, which has been observed in some systems, and found none. They next assessed fidelity, and identified two Cas13 variants that exhibited no tolerance for mismatches between the crRNA and target sequences. They then generate and test three Cas13 variants to expand potential applications: 1) a nuclease-dead version that they demonstrate can be used for RNA pull downs, 2) a mitochondria-targeted version capable of efficiently knocking down mitochondrial transcripts, and 3) an ADAR2 fusion for targeted RNA editing. Finally, they build on these findings to move in vivo. The authors generate multiple Cas13 Drosophila lines and demonstrate efficient knock down of target transcripts that recapitulate known mutant phenotypes. Overall this work is very thorough and will be of great interest to the entire Drosophila research community, as well as researchers in other models seeking to adapt Cas13 for in vivo RNA targeting. The authors establish valuable toolsets and define the parameters for optimal Cas13-based targeting. Their comprehensive analysis of Cas13 variants with widely varying activity levels also provides a valuable resource for fine tuning transcript knock down. Concerns are minor and can largely be addressed through changes to the text and figures.

Specific comments:

1. All statistical tests need to be described and justified (e.g., are data normally distributed). Figure 5 lacks statistics.

We have added a section named “statistical analysis” in the methods section. We have also double- checked the text, and revised relevant result sections and figure legends accordingly.

2. A bioRxiv paper (Buchman et al.) describing the use of the CasRx in flies was posted in April. They observed significant toxic effects from expressing Cas13 alone. Experiments addressing potential toxicity in flies should be included. It would also be ideal for the authors to cite this work and address in their discussion. Note that I don’t think it affects the significance or novelty of this manuscript since Buchman et al. only investigated one Cas13 variant with much less success due to general toxicity and low efficiency.

Thanks for bringing our attention to this paper, in the meantime it has been published in the CRISPR journal (https://doi.org/10.1089/crispr.2020.0018). In their study, Buchman et al. tested the original version of CasRX isolated from *Ruminococcus flavefaciens* XPD3002 (Konermann et al. 2018). We also generated transgenic versions based on this CasRX version. Our first attempt was to insert the CasRX transgene onto the third chromosome. This resulted in very sick flies, even when only one copy of the transgene was present. We were unable to keep the stock in our collection due to the high lethality (as such, we don’t have proper survival charts for this line). Our second attempt was to insert the CasRX transgene on the second chromosome. This line was viable and you can see the data in the picture below (aka the new Figure S8). In brief, we still observed 100% lethality in homozygous CasRX populations. The heterozygotes, however, were fine. Similar to CasRX, we also observed low survival rates in a transgenic line homozygous for PspCas13b. Due to the high lethality in the homozygotes, we decided to not further investigate the CasRX and PspCas13b transgenes. By comparison, the CasFX and CasFB transgenes we generated for this study showed only moderate drops in survival even in homozygous animals, and heterozygotes were indistinguishable from controls. We included the survival rates as the new Figure S8 in the manuscript, and we also mentioned the Buchman et al. paper in the discussion section.

3. For the Cas13 pull down experiments, the argument against also looking directly at mRNA (lines 293-298 ) is not compelling and the authors should show both target mRNA and protein. The text and figure should also make clear that the HA tag on Cas13 was used for pull down.

We agree that this is a valid point. We have added two subfigures S5A and S5B to address this concern. Figure S5A shows that dCasFX can bind target mRNA alone, and that the results are comparable for all nine crRNAs. We were curious, though, whether we would find that in the presence of IRP1A, we would see competition between dCasFX and IRP1A if the crRNAs would correspond to sequences that are in the vicinity of the IRE (the binding site for IRP1A). The results are shown in Figure S5B and indicate that the two proteins do compete, since the presence of IRP1A causes a ~4-fold reduction in immunoprecipitated mRNA (this result nicely complements the data in Figure 4E). We also rephrased the text accordingly.

4. In the experiments targeting multiple transcripts (Figure 6G), did the authors also conduct qPCR? It would be very interesting to quantitatively compare the efficiency of knockdown to single targeting experiments.

We have added the Figure 6H to address this question. It appears that the efficiency of targeting two genes simultaneously was comparable to target the genes individually (at least for these two genes, *dib* and *IRP1A*)

5. Lines 423-425 are confusing. What does ‘sequence deviations’ refer to? Do these changes go beyond nearby A->I modifications? This should be clarified and, if the answer is yes, addressed.

“Sequence deviations” refer to nearby A --> I modifications. There were non A --> I mutations at a low frequency (approximately 1.6% - 2.4%) across all samples. However, the non A--> mutations were also detected in control samples, also at a low rate. We reason that these sequence deviations can be a result of random mutations during transcription or errors when processing extracted RNA for sequencing (reverse transcription, sequencing, etc.). Overall, sequence deviations have been reported in the original REPAIRv2 study (Cox et al. 2007). In that study, Cox et al. also reported a low frequency of non A --> I modifications in REPAIRv2 (approximately 2.5% - 3.2%) or in REPAIRv1 (also 2.4% - 3.5%). Also, consistent with Cox et al., the sequence deviations rate in FREPAIRv2 was also lower than REPAIRv1.

In any case, we altered the wording only slightly to improve clarity without adding more text.

6. Overall the writing was very clear and easy to follow. However, the text could benefit from trimming. The introduction and discussion/conclusion in particular can be focused to hone in on key points and remove redundancies. The results section on the REPAIR construct can be shortened a lot.

We have polished the text throughout, improved clarity and condensed the text overall. We have also shortened the relevant REPAIR-related sections.

7. The authors do not demonstrate that the addition of NLS or NES sequences successfully modulate the subcellular localization of the Cas13 variants, limiting their ability to conclude that activity is independent of subcellular localization. This caveat should be addressed or complementary imaging data should be provided (Lines 193-196 and Figure S3).

We have included a subfigure S3F that demonstrates that the NES/NLS constructs work as expected, at least for CasFB5 and CasFX4 (the most highly efficient Cas13 variants). We have also briefly explained how the constructs were generated based on previous studies. We built these constructs based on prior Cas13 studies from other labs as well from similar constructs for CRISPR/Cas9 we generated in our own lab. The subcellular localization of corresponding LwaCas13a, PspCas13b and CasRX constructs have been tested in plant cells (Mahas et al. 2019).

8. While dCasFX could in principle be used to determine subcellular localization of target RNAs, the authors haven’t shown this and should temper statements accordingly (Lines 320-321 and 519).

Thanks for pointing this out. We only meant to refer to this as a potential application of dCasFX that would require proper modifications and changes in the experimental design. We have toned down our statements accordingly.

9. The Cas13 variant naming is a bit confusing. It might be helpful in the paragraph starting on line 112 to explain why d-subtype variants are named with an X.

“CasRX” was coined by Konermann et al. for the Cas13d ortholog isolated from *Ruminococcus flavefaciens* XPD3002 in order to distinguish from Cas13d orthologs in other species {Konermann et al., 2018, #15651}. Since we generated the fly-optimized versions of CasRX, we refer these versions as CasFX as a reminder of its origin. We have added this explanation to the main text.

10. There are some missing references:

Lines 89-92: The original 2013 Drosophila CRISPR-Cas9 work from several labs is omitted.

Lines 115-117: The references for the relevant mammalian cell culture should be included here.

Fixed. Citations have been added.

11. Occasionally, unsupported or overly strong statements are made about various related technologies, e.g. meganucleases, zinc finger nucleases and transcription activator-like effector nuclease on lines 62-65; RNAi and CRISPRi on lines lines 80-81; and CRISPR-based techniques on line 92.

Fixed. We have rephrased the statements and provided supporting citations.

12. The graphical representation of lethality rates from Table S3 might be more informative in place of or in addition to the phenotypic imaging provided in Figure 6. The images in these panels may be better suited as supplementary data.

The purpose of the phenotypic images (Figures 6A-C, 6G) is to provide a succinct overview of the effect of the genotypes and dietary supplementation on survival. Table S3, on the other hand, provides detailed information about survival rates at each developmental stage. We have considered replacing the phenotypic figure with the survival graphs. However, we realized that this would amount to 40 individual graphs, which we felt would make Figure 6 look too busy. After consideration, we decided to make the survival graphs into a separate supplementary figure (see new Figure S7, which we also pasted below), which are based on Table S3.

13. On Line 541, the modifier ‘real’ should be removed.

Fixed

14. Line 43 should read CRISPR-associated proteins instead of CRISPR-associated proteins.

Sorry, but since both phrases are the same, we are not sure what you meant.

Many thanks for the great suggestions!

Reviewer #2:

The work by Huynh et al. entitled " A versatile tool-kit for CRISPR-Cas13-based RNA manipulations in Drosophila" demonstrates the interrogation of Cas13 variants to identify a potent variant, Cas13d, for targeted knock down of RNA transcripts, in vivo and in cell culture.

In this study the authors tested 40 Cas13 variants of the four subtypes of the type VI CRISPR system. Cas13d exhibited around 82% efficiency in knocking down the target transcripts whereas CasFX4 variant showed 90% knock-down efficiency. Interestingly, their data have confirmed a recent report that the nuclear of cytoplasmic localization of the Cas13 variant does not seem to influence the efficiency of targeting the RNA transcripts. And consistent with studies in mammalian and plant systems, Cas13 variants see to lack collateral activities in vivo.

Furthermore, CasFX4 and CasFX8 exhibit low off-target activities. And these variants were tested for RNA knock-down, RNA- recognition and RNA editing as well as targeting mitochondrial RNA transcripts. The authors have nicely shown different applications of the CRISPR- Cas13 system beyond RNA interference which will be key for the future applications of D. M research. This toolkit will definitely be useful to the community.

Similar toolkits are highly needed for other eukaryotic species to expedite basic and translational research activities.

The manuscript is very well written, and the topic is timely.

Thank you!

This manuscript builds on prior work from this lab on the use of CRISPR systems for the manipulations of the Drosophila genome. And all methods are relevant to this research project and to the aims of this study. Most importantly, the conclusions are supported by the experiments and the data. If the authors produce a tool-kit, this implies that the reagents will be able from Addgene or similar platform for distribution to the Drosophila labs. I think the authors need to include a statement on how they are planning to share these reagents.

We have added the requested information. We have included a statement on how we are planning to share these reagents in the “Data availability” section.

Although the work demonstrates the feasibility of using CARISPR-Cas13 systems specifically CasRx for different RNA manipulations, it is very useful for the community and such toolkits for other systems will be equally eligible to receive similar attention. This manuscript is of great interest to D.M research labs and will impact the basic and applied research in this model system

**Second round of review**

**Reviewer 1**

Huynh and colleagues have done an excellent job of addressing most of my concerns. However, the authors describe using t tests rather than analysis of variance with post hoc tests for multiple comparisons (e.g., Fig. 3). All multiple comparisons should be reanalyzed with appropriate tests. Figures should also make clear what comparisons are being made as this is not always obvious (e.g., Fig. S5A and B). In addition, most journals now strongly encourage the use of scatter plots over bar graphs, and I highly recommend the authors make this change (with apologies for not noting it in the first round of review).

Minor point:
1. The legend in Fig. S5 should refer readers to Fig. 4E where the crRNAs numbers are explained.

**Authors Response**

**Point-by-point responses to the reviewers’ comments:**

Reviewer reports:

Reviewer #1: Huynh and colleagues have done an excellent job of addressing most of my concerns. However, the authors describe using t tests rather than analysis of variance with post hoc tests for multiple comparisons (e.g., Fig. 3). All multiple comparisons should be reanalyzed with appropriate tests.

Fixed. After consulting with a statistician, we have reanalyzed all data depicting multiple comparisons using Dunnett’s post hoc test (Figures 3, 4H, 6E, S3, S4) and Tukey's range test for Figures S5A,B for multiple pairwise comparisons. Figures were updated accordingly.

Figures should also make clear what comparisons are being made as this is not always obvious (e.g., Fig. S5A and B).

Fixed, we have added a statement in the statistics section (Methods) as well as in the figure legends.

In addition, most journals now strongly encourage the use of scatter plots over bar graphs, and I highly recommend the authors make this change (with apologies for not noting it in the first round of review).

Done. Scatter plots have been added to bar graphs.

Minor point: 1. The legend in Fig. S5 should refer readers to Fig. 4E where the crRNAs numbers are explained.

Fixed. Thanks!
